# Supplementary material for: Age- and ApoE Genotype-Dependent Transcriptomic Responses to O3 in the Hippocampus of Mice
Source: Int J Mol Sci. 2025 Mar 7;26(6):2407. doi: 10.3390/ijms26062407 (PMC11942628; doi:10.3390/ijms26062407)
Supplement: Supplementary file 1 [file ijms-26-02407-s001.zip › Supplementary Table S1 qRT-PCR results of selected genes.pdf]

Supplementary Table S1. QRT-PCR results of selected genes to validate the RNASeq results

Gene	qRT-PCR Fold change E4 Vs E3 (17M O_3)	*p*- Value	*RNASeq* Fold change E4 Vs E3 (17M O_3)	*p*- Value
Sfrp5	4.06	0.05	6.5	1.08E-04
Folr1	2.04	0.05	16.5	1.65E-06
Bok	2.85	0.02	4.6	0.02
NeuN	4.46	0.04	NDE	na
DC	1.33	0.59	NDE	na
Twist	2.44	0.29	3.3	0.05
Pon3	3.13	0.26	4.4	4.14E-04
Klotho	16.5	0.17	5.5	6.36E-04
Barhl1	2.30	0.38	12.2	0.01
Casp1	-0.203	0.36	-2.9	2.17E-04
Apaf1	-0.81	0.29	-2.0	8.83E-04
