# Supplementary material for: Age- and ApoE Genotype-Dependent Transcriptomic Responses to O3 in the Hippocampus of Mice
Source: Int J Mol Sci. 2025 Mar 7;26(6):2407. doi: 10.3390/ijms26062407 (PMC11942628; doi:10.3390/ijms26062407)
Supplement: Supplementary file 1 [file ijms-26-02407-s001.zip › Supplementary Table S2a List of upregulated genes in the Venn diagram.pdf]

Supplementary Table S2a. List of upregulated genes of the three-factors (genotype, age and treatment) and the intersections in the Venn diagram

Factors/intersections	Upregulated Genes
**Genotype E4 =1490**	Ptprh, Itgb4, Ahnak, Pdyn, Gm1821, Erap1, Vwa3a, 4930412C18Rik, C130060K24Rik, Cd82, Gm17750, Wfdc2, Gm10687, Nme8, Il25, Col8a2, Aldh1a1, Tlx2, Slc4a5, Sema3a, Sp8, Ocln, Carns1, Plp1, Rbp1, Ankrd66, Ankrd63, Zfp647, Nkx6-2, Gria1, Ezr, 1700010I14Rik, Fgf10, Slc5a8, Trim21, Angptl2, 4930427A07Rik, Cflar, Polr1a, Fbxo32, Nt5e, 4932411E22Rik, Rnf152, Phex, Gm16150, Cdkn1c, Kcnk5, Sfrp1, Ccdc175, Tmprss11a, Cd63, Rdh5, Daw1, S100pbp, Anxa4, Tgfa, Antxr2, Fign, E030010N08Rik, Pcolce2, Xaf1, Gm26531, Spag6, Ribc2, Hspb6, Mcoln3, Clec3b, Slc12a8, Ptgr1, Ccdc89, Wipf3, Rhog, Ggh, Slpi, Mettl24, Fzd7, Htr2c, Slc16a6, Osr1, Lats2, Anxa8, RP24-467O4.1, Cyp4f17, Nod2, Lrrc71, Dlec1, Rassf8, Ccdc135, Tmem47, Cdca7l, Myl12a, Sik1, Arhgap28, Dock5, Atp4a, Ttc16, Gm973, Wdr86, Slc14a2, Krt23, Gm14052, Tgtp1, Arhgap12, Sh3rf2, Tgfb2, Gpr37, Sox2ot, Upb1, Antxr1, Fam189a2, Cyp27a1, Tgfbr3, Car9, Gpr149, Mlf1, Gjc2, Opalin, Vwa3b_11, Mrgprh, Lrrc34, Tcea3, Cd59a, Krt15, Eef1a1, Zfp92, Atp2b3, Zbtb20, 2610015P09Rik, Drd3, Nipal4, Cpq, Gmnc, Wbscr17, Gm12762, Ccdc11, S1pr5, Klhl31, Zfp423, Defb11, Cnpy1, Sil1, Serpinb1b, Hs3st1, Cdh3, Gm6104, Plekhb1, Dnajb13, Slc39a4, Strip2, Galnt6, Tbcel, Mxra8, Slco1a5, Gm7173, Sdc1, Ifi44, Kcnq5, Gm16638, Syndig1l, Prlr, 2610028H24Rik, Ebf3, Rhod, Capn6, Rtp4, Hist1h1c, Fam60a, RP23-8J15.5, A2m, Hmgn5, Sh3bgrl, Gad2, Cdr2, Avpr1b, Cd24a, Tmem27, Pdk4, Serpina6, Esrp1, Ppap2c, Zfhx4, Sult1c1, Slc5a7, Igf2os, Stard13, Dusp6, Epha4, Ncl, Zfp820, Tuba1c, Fzd5, Pcca, F5, Apol9a, Gata3, Rnf128, Nutm1, Klhl4, Slc18a2, Isl2, Oit1, Gm14051, Brwd3, Maats1, 3300002A11Rik, Mospd1, Prob1, Irak4, Olfr1507, Rbp2, Fblim1, Nlrp5-ps, Rrh, E230013L22Rik, Orai2, Cd4, Plscr2, Rilp, Lhb, Galm, Epha7, Col8a1, Slc37a2, Tmem167b, Itpripl1, Mgl2, Ankub1, Wfdc6a, Dynlrb2, Cyp4a12b, Map3k19, Col6a5, Fam204a, Gm17334, Wscd2, Zfp677, Cyld, Arhgef5, Bmp7, Gm26580, Krt19, Mgst1, Pi4k2b, 1700001C02Rik, Pdpn, 2810025M15Rik, E230008N13Rik, Heg1, Sowaha, Fam209, E530001K10Rik, Slc13a4, Smc4, Il13ra1, Lpl, Lhx9, Ppp1r1b, Tspan4, Glis3, Emilin2, Styxl1, Ppfibp2, Pih1d2, Grin2b, Emp1, Lrp6, Slc12a2, Nod1, Tmem88b, Slc24a5, Folr1, Gm14275, Cers2, Gdnf, Ifitm3, Irf7, Wls, Golm1, 2210016F16Rik, Slc28a3, Tob1, Rnf43, Arfip1, Tuft1, Ccdc122, Rasd1, Gm1110, Calml4, Nudt12, Fam19a1, Postn, Acnat1, Trim36, Prdx4, Gm14230, Kif6, Pdgfd, Zfp521, Gm1661, Gatm, Slfn9, Wfdc17, Rarres2, Gpr63, Cacng5, Amotl1, Col4a3, RP23-8J15.7, Prr18, RP24-91A16.3, Gtse1, Fhad1, Pcolce, Sntg2, Gm6206, Sall4, C6orf183, 5430417L22Rik, Aqp1, Kcne4, Col25a1, 1700026D08Rik, Hydin, 4930429F24Rik, Lrguk, Lgals1, Hist1h2bc, Perp, Hacl1, Aim2, Shox2, Lrrc23, Traf1, Zeb2, Cutal, Gsn, Tmem212, Ttyh2, Ctf1, B3galt5, Ifit3, Ptk2b, Cldn14, Il17rb, Adgb, Ermn, Lpin3, Pla2g5, Acaa2, Gbx1, Dmkn, Homer3, 4933406P04Rik, Zfp386, Myo3b, Npb, Tor4a, Thsd4, Mbp, Gm13617, Gm5815, Capsl, Kcne2, Iah1, Trp73, Asb10, Stxbp4, Cep41, Rgs14, Clec12a, Pcdhb1, Rab11fip1, Erlin2, Catip, Rsf1os2, Sytl3, Adamtsl4, Hrasls, Nid2, Dpyd, Olfml2b, Mog, Apold1, Sgk3, Pbxip1, Zic4, Asah2, Prkcq, Yme1l1, Ifit1, Met, Slc16a12, Ager, Gm2a, Adra2b, Dthd1, Lipa, Slc22a8, Zhx2, Stk33, BC051019, Akip1, Ippk, Lrrc10b, Gm15906, Ccdc108, Gm16880, Ak7, Dach1, Cdh26, Stim2, Glb1l2, Styk1, 1500015O10Rik, Slco1c1, Tekt3, Glp1r, Wfikkn2, Apobec2, BC021767, Serpinb8, Serping1, Aga, Vegfc, Ajuba, Rassf10, Csf1, 9930111J21Rik2, Bcar3, Abca4, Fbn1, Fgf7, Gm14327, Anxa2, Cgnl1, Fcgbp, Cldn9,

Marveld3, Tat, RP23-69L13.5, Tacr1, Spata18, Lpar3, Ptch2, Best3, Il5ra, Gtsf1l, Trim59, Tmem215, Xirp1, Epn3, Baiap3, Il12rb2, Oscar, Elf4, Tbxa2r, Plek2, Kif9, Ptgs2, Fam46c, Apobec1, Cd55, Sec14l1, Rpa1, Magel2, Thbs1, Ptgis, Dcdc2a, Ptprq, Ccdc40, Mavs, Tac4, Lrrc46, Ptpn3, Insc, Ggt5, Tgfbi, Dmrt3, Spint2, Tram2, Gm16043, Musk, St6galnac2, Wnt11, Ifi27, Iqck, Fam83e, Gm9762, Gm26645, Pls1, Pcp4l1, Aebp1, Cpt2, Mut, Loxl4, Myb, Gm11992, Dnah11, Gzmm, Amigo2, Rbm47, Nr3c2, Slc10a4, Tec, Evi2a, Klk6, Inadl, Tctex1d4, 2610305D13Rik, Rbms3, Acap1, 9930014A18Rik, Ankk1, F11r, Sap30bpos, Car12, Efcab1, 1700009P17Rik, Tc2n, Cort, Gm12695, Gm13142, Slc26a3, Islr, Maml1, Abca1, Aox1, Bmp4, Mvp, BC026585, Zfp951, Spag17, Casq2, Vangl1, Prdm16, Nphp1, Zic1, Ctxn3, Slc39a12, Tmem98, 4932443I19Rik, Dact1, Pmp22, Fam198b, Trim56, Spata6, Dbndd2, Gm14418, Nbl1, Ddx58, Cndp1, 1700112E06Rik, Cldn3, Smim22, Anln, Maml2, 1700012B09Rik, Creb3l2, Cd9, G6b, Chsy1, Cep128, Frmd4b, Col4a4, Hey2, Trpm3, Cdhr3, Emb, Tmem190, Flnc, Prkg1, Tbc1d2, Prr5l, Abcc4, Npffr1, Unc5b, D7Ertd443e, Mal, Gab1, Ppp1r32, Zfp36, 1700101E01Rik, Prelp, Stk39, Tppp3, Mpp7, Gm3267, Slit2, Zfp819, Dse, Cdnf, Serpina3n, Gsc, Enpp6, Ppp1r36, Vim, Creb3l1, Ulk4, Gbx2, Ttc12, 1700028P14Rik, Gm26813, Ak9, Dnaaf3, Gm17455, Ano1, Fgf3, Tiparp, Enpp2, Syde2, Smad6, Gabrp, Kcnmb1, Actr2, Sox10, Zc3hav1, Gm4477, Mthfd2, Plekha2, Kiz, Itpkb, Baiap2l1, Ntn4, Prkcd, Slc2a12, Atp7a, Slc1a5, Tmem237, Lrmp, Mmp15, Matn2, A730049H05Rik, Ubxn10, Rasl11a, Vit, Eif2ak2, Gm10224, Plekhg1, Odf3b, Tns4, Apol9b, 2410004P03Rik, Rap1a, Parp9, Wasf1, Cebpd, 1500012F01Rik, Oasl2, Lipo1, Chrnb4, B4galt1, Zfp536, Fam46a, Tmprss5, Pde3a, Aspa, Sgms2, Slitrk6, Ppp1r16b, Plcl1, Duox2, Mob3b, Lrrc48, Spef2, Mansc4, Sult1c2, Gm853, Nuak2, Rest, Armc4, Ttr, Pkp2, Gm15713, Cldn19, Fam47e, Mag, Ctnnal1, Ccdc121, Trpv4, Tmc4, Litaf, Rbpms2, Cecr2, Spata17, Frem1, 4932438H23Rik, Chn2, Gimap6, Foxi1, E230019M04Rik, Mfap4, Kat2b, Sntn, Gm9234, Stra6, Rnf13, Gm2044, Anxa11, Elf1, RP24-73K7.1, Plekhh1, Mboat1, Tspan2, Col4a6, Col4a5, Pabpc5, Bdnf, Uncx, Sh3tc2, Kank2, Rassf4, Pip5k1b, Fgfr2, Ehhadh, Ggt6, Gm16090, Zhx1, Igtp, Rarb, Prps1l3, Kif27, Ikbip, Sgk1, Dnah1, Krt8, Scube3, Fam167a, Sln, Tmem106a, Wdr96, Utp14b, Bbox1, Kl, Gm6472, Iigp1, Gbp11, 4930529M08Rik, 3110001I22Rik, Bdkrb1, Gbe1, Ebf2, Efcab10, Pde8a, Vwa5b1, Rffl, Chrna3, Creb5, Slc23a2, Vcam1, Neurog2, Mmp2, Ttc25, Cnp, Krt18, Ifit2, Lhx8, Wnk4, C030048H21Rik, Ccdc114, Wnt3, Wnt9a, Iqca, Bmp6, 4833427G06Rik, Slc31a1, Nfe2l2, Pld5, Hfe2, C1ra, Arhgap33os, Hist1h4m, Kirrel2, Tcam1, Gh, Rragd, Tor3a, Il17re, Slc12a4, Slc35d3, Gjb1, Gm12326, Hist1h2be, Hist1h4h, Gm11847, Tmem170, Fzd6, Wdr63, Ttc18, Iqgap2, Prps2, Txnip, Efemp2, Cpt1a, Fam115c, C2cd4b, Onecut3, Armc3, 2310007L24Rik, Slc38a3, Gpc3, Eps8l2, Sgk2, Six3, Ccdc153, Tmcc3, Spata13, Slc44a3, Trim30a, St18, Syt9, RP23-5F21.1, St8sia6, Plbd1, Gm16160, Isl1, Cilp, Gm5860, Cldn11, Rgs22, Rab20, Dab2, Gm13283, Tekt1, Phactr2, Ifitm2, Myo1d, Stxbp3a, 1700013F07Rik, Ccnd3, Dnah3, Cxcl5, Hspa2, Bfsp1, Drd2, Snrpc, Stoml3, Trim30d, Zfp52, Ankrd65, Msx1, Serpinb9, Klhl13, 4932418E24Rik, Slc25a13, Sema6a, Klf3, Cpm, Ddc, Csrp2, Lhx1, Fam161a, Rtdr1, Slc16a1, Vcp-rs, Prrg1, Espl1, Zbtb7c, Vamp8, Tspan6, Lingo3, Sema3b, Gpr50, Plekhg3, Chat, Mc3r, Ccdc113, H2-Aa, Gm26747, Clic4, Slc5a1, Gm4524, Rbm3, 4933426M11Rik, Ntrk1, Col9a3, Atf3, Gm26684, Asb18, Fkbp14, Thumpd3, Pcdh20, Katnal2, Pla2g4a, Sema3e, Plcb4, Tm4sf1, Hspa5, Kynu, Ispd, Hpdl, Jph1, Fam216b, Dnaic2, Acot6, C3, Vat1l, Mr1, Chdh, Fsbp, Gm20632, D630039A03Rik, Tcfl5, 9130023H24Rik, Atp11c, Msx2, Slc16a8, RP23-375H15.1, Mapk15, Igf2bp2, Gstm7, Bok, Bsg, Arrdc3, Igsf5, Arpc5, Tjp3, Wdr72, Ttc21a, Mobp, Scn5a, Slc35g1, Sstr5, Slc29a4, Zic3, Irf1, Mb, Steap2, Steap1, Ccdc170, Iyd, Cd109, Fkbp9, Lrrc1, Corin, Zfp719, Agbl2,

Fam126a, Rhoj, A530053G22Rik, Bhlhe23, Hspb1, Efhc1, Sox1, Frzb, Ctnna1, Fem1c, Pafah2, Vipr2, Heph, Oprk1, Lhx5, Wnt8b, Gm101, Syngr2, Nkx2-2, Itpka, Sipa1l3, Parp14, Snrpd1, Slc12a7, Pltp, 1700007G11Rik, Cldn2, Tnnt1, Six3os1, Dppa5a, Nfatc3, Rd3, Rsph3a, Ccser1, Thnsl1, Kntc1, Foxn2, Ntf5, Acacb, Gpr4, Hrasls5, Gstm2, Slc26a7, Igfals, Dlx3, Dpep1, Fap, Diap3, Olfm4, RP23-32P19.3, Erbb2ip, Thbs4, A730090H04Rik, Acss3, Parp4, Clcnka, Npr1, Wdr52, Irx3, Irx5, Dnah10, Slc22a18, Lamp2, Nkx2-1, Sost, Inf2, Rp2h, Lrriq1, Spag16, Gm5602, Tspan18, Smco3, Sox3, Gm13577, Tpd52l1, Tead1, Nhs, Gm6548, Insl6, Ccdc146, Coch, Fam84b, Timm8a1, Cul4b, Pdia4, Iqcg, Adora2a, Sptlc3, Wdr93, Vpreb1, D630024D03Rik, Dynlt1f, Bhmt, Mettl18, Mdfic, Zfhx3, RP23-281H4.7, Spry1, Igsf1, Rnf122, Myof, Igfbp2, Pon3, Prkch, Filip1, 4930408O17Rik, Lrrc2, Tsku, Sowahc, Gjc3, Slc24a3, Ccdc55, Abhd2, C130074G19Rik, Dnah6, Plagl1, Cd164, Gm10714, Ttll6, Nt5dc2, 1700007K13Rik, Ccdc37, Shroom3, Hp, Lgals3bp, Rbm46, Ins2, Lrrn2, Otx2os1, Bicc1, 4430402I18Rik, Gm13251, Syne3, Nek5, Prr32, Tmem220, Ap1s2, Fuca1, RP24-531B21.1, Rab40b, Pde7b, Eci3, Fam183b, Ttc23, Synm, Zfp551, 2010001K21Rik, Ly6g6f, Nek11, Rassf9, 9530056K15Rik, Kcnh1, Cpne4, Acpp, Bex1, Fli1, Tmem125, I830012O16Rik, Tmc3, Col6a3, Zfp189, Ldlrap1, Npy2r, Acot1, Sod3, A230065H16Rik, Actn2, Ccdc78, Ripk4, Ddr2, Enkur, Oca2, 2010300C02Rik, Foxj1, Bst2, Rgs3, Gpr110, Prokr2, Gm26812, Otx2, Lum, RP24-187D4.1, 4930522L14Rik, 5430403G16Rik, Notch2, Sytl4, Prss23, Foxp2, Lpar1, Crb2, 1810019D21Rik, Prrg4, Eps8l1, Shisa6, Piezo2, 45179, Gm2991, Ucp2, Ddr1, Pou4f2, Itgb6, Cachd1, Xrcc4, Zic2, Pnlip, Lrp10, Gm21971, Vsig10, Wdr65, Sh3d19, Mycbpap, Acox2, Cacng8, Gm16201, Ugt8a, Gm20501, Adam12, Hdc, Vav3, Th, Crhr2, Inmt, Zfpm1, 1600029I14Rik, Hspa1b, Ppp1r3b, Casc1, Tgfbr2, Gm8787, Ssfa2, Pilrb2, Tll1, Galnt12, Gabrr2, Nt5dc1, 4921524J17Rik, Leprel1, 1700003M02Rik, Nqo1, Frmpd1os, Adamts4, Wdr49, Erich2, Mest, Tmprss6, Nkx2-9, Chia1, Snx9, Dgkb, Myrf, Maml3, Fa2h, Rd3l, Wdr16, Gulp1, Igf2, Cckar, Elovl7, Tmem108, Slc16a2, Avpr1a, Ccdc162, Marveld1, Tep1, Hist2h3c2, Hfe, Psme2b, Penk, Zmynd10, Ehf, Adamts5, Adamts1, Tmem184a, Ecm2, Cald1, Lmx1a, Nuf2, Clic1, Gm11517, Gm26905, Ifi27l2a, Dnajc3, Cox8b, Bdh2, Slc6a19, Gng8, Lpar6, Zap70, Erbb2, Edar, Serpinb6b, Ak8, 1700026L06Rik, Itfg3, Pgm5, Tspo, Ankfn1, Fam187b, Gm13807, Icos, Tnfaip6, Slc16a9, Heatr2, Prl, Zfp185, Tcn2, Gm16045, Zfp518a, Ctsc, Ccdc96, Elf3, Epb4.1l4a, Scg2, Olfml1, Gprc5c, P4ha3, Plxnb2, RP23-132E8.2, Ccdc81, Iqub, Trp63, Cldn1, Srl, Arsg, Car13, Gm27031, Mfsd7c, Ttll9, Tpt1-ps3, Gpr98, Pgpep1, Cnksr3, Palmd, Dnah12, Efcab7, Cenpi, Gng11, Bnc2, Frmd8, Ccdc60, 1700007K09Rik, Tnni1, Man1a, Lamb1, Pifo, Serpina3m, Pou6f2, Echdc3, Rsph9, Cyp39a1, Olfr570, 3930402G23Rik, Cdk1, 1700040L02Rik, Wdpcp, Serpinh1, Npr3, Nfia, Epcam, Prdm12, Lrrc18, Nqo2, RP23-103C13.2, RP23-69L13.3, Peg10, Cd209b, Acot2, Hspg2, Gas2l2, Ace, Col17a1, Loxl1, BC067074, Gpx8, Snhg7os, Dgkg, Adra1d, Gm11889, Wdr78, Usp18, C330011M18Rik, Sostdc1, Il16, Fgl1, 9130019P16Rik, Iqcd, Pvrl3, Rdh10, Clca2, Gbp3, Zfp758, Ostf1, 8430408G22Rik, Mfrp, Acot5, A230009B12Rik, Hist1h2bg, Sulf1, 2700046A07Rik, Serpina3e-ps, Dock10, Acad8, Plin5, Rsph4a, Sp9, Smad7, Myo5b, Morn3, Fam166b, Zc2hc1c, Lect1, Ttc30b, Lrp5, Tmem63a, Gabra5, Cab39l, Bambi, Arhgdib, Rerg, Pik3c2g, Dtx3l, Ctsd, Rsph1, Rnf182, Lrp2, Slc43a3, Rpl3-ps1, Cdc20, 1500015L24Rik, Rnf138rt1, Htr1d, Lrrc36, Chaf1a, Got1l1, Gm12474, Crygn, Nme5, Clic6, Slc16a4, Tsnaxip1, Scn11a, Dnah2, Twist1, Mns1, 4933407I08Rik, Rfx2, Gm867, Zic5, Lhfpl1, Samd3, 4930448N21Rik, Smim18, A430105I19Rik, Gm7701, Gstt3, Eno4, Map3k15, C1galt1c1, Dnali1, Topors, Igfbpl1, Bcas1, 4931429I11Rik, Clmp, RP23-19M11.1, Nrap, Plce1, Gm27202, Fstl5, Slc26a2, Trim7, Lbp, Btd, C1s1,

	Car14, C1rl, Sfrp5, Gm10357, Tlr2, Ccdc109b, Zfp419, Zfp804a, Caps2, Isg15, 4921536K21Rik, Stc2, Fbln7, Tfcp2l1, Epb4.1l5, Dcdc5, Col18a1, Ackr4, Lepr, Stmnd1, Smpdl3a, Stk32a, Mettl20, Nxn, Col9a1, Ephx1, Gpr151, Fzd4, Ccbe1, Smim5, Cyp4f16, Snx33, Gm10643, Gm12144, Gyltl1b, Irgm1, 1700066O22Rik, Ccdc33, 4930451C15Rik, Efhb, Cpn1, Pnp2, Gm15510, Lhx1os, Serpina9, Kcnj13, Lgals3, Arhgap24, Ccdc42b, Shisa3, Zfp229, Pon1, Myh14, Ttc29, Tmem72, Lama5, Frk, Fbxo5, Sorbs3, Athl1, Ptafr, Apod, Ooep, Ddx60, Fam107b, Oxt, Rps27a-ps2, Loxl2, Slc4a2, Tbc1d8b, Serpinb1a, Bcas1os1, Spag8, Erbb3, Cdc40, Qprt, Hhat, Enpp1, Fgf16, Magt1.
Age 17M=760	Itgb4, Clec5a, Wfdc2, Slc4a5, Plekhd1, Tspan11, Smpdl3b, Carns1, Gm13112, Nkx6-2, Epsti1, Hcls1, Fbxo32, Nt5e, Pisd-ps1, Itgb2, RP23-329F7.2, 4930509E16Rik, Gm26529, Cd84, Rdh5, Fign, Cd48, Xaf1, Zbtb8a, Reep6, Dock2, Slc2a4, Rreb1, Pou4f3, Rhog, Cnga2, Gpnmb, Rbpjl, Cbln3, Itgax, Lyve1, Cldn22, Gm26533, AI115009, Pla2g3, Cd163, AW112010, Nrl, Wdr86, Ifi47, Tgtp2, Fmo2, Isg20, 5330438D12Rik, Eva1c, Slc11a1, Vil1, Cyp27a1, Galnt5, En2, Ctla2a, Hs3st3a1, Mpeg1, Pydc4, Zan, Ly86, Stamos, Rpl37rt, Plin1, S1pr5, Sphk1, Serpinb1b, Cdh3, Gsdmd, Ifi44, H2-Oa, Acr, Ada, Icosl, Rpgrip1, Rtp4, Gch1, Ptgs2os, Syt6, Slc45a3, Avpr1b, Rcsd1, Rasgef1c, Prdm8, Hapln2, 9130208D14Rik, 0610040B10Rik, Ccrl2, Apol9a, Gm10101, Gm21743, Gm15892, Wfdc1, Arid3c, Igsf9, Cd37, Gm10334, Rgn, Egln3, Nlrp5-ps, Car4, Gm10288, Alox8, Gucy2e, Lhb, Pabpn1l, Gabrr1, 0610009L18Rik, Col8a1, Srgn, Dynlrb2, Tmco4, Pcdhb2, Hmx1, Gm18355, Cd300lb, Dapl1, Pcsk4, Cabp5, Etnppl, Olfr287, Abhd12b, Gm6792, Clca6, 4930581F22Rik, Samd13, Ccdc169, Irf8, Folr1, Nanos2, Osmr, Cdc42ep5, Ifitm3, Drd4, Cdhr5, Irf7, Igkc, B2m, Gna15, Gm13868, Crybb1, 2610034M16Rik, Neurl3, Col2a1, C130026I21Rik, Slfn9, Gm9887, Col4a3, Rac2, Hba-a2, Hba-a1, Batf3, Aqp1, H2-M3, 4930429F24Rik, Gm16271, Rtp1, Shox2, Lag3, Lrrc23, Gm8995, Tmem212, Gm2415, Ceacam1, Ubash3a, Ifit3, Ly6a, Arrdc2, C1qa, Pld4, Aldh1a2, Gpr115, Olfr550, Hs3st3b1, Piwil2, Irx1, Homer3, Oas2, Nlrp3, Fbxo48, Hhatl, Fhl4, Tnfsf13, Kcne2, Vsx2, Dffb, Herc6, Pglyrp1, Siglec1, Oit3, Crygb, 1810010H24Rik, B230312C02Rik, Pcdhb9, Rgr, Cdhr1, Prss56, Apold1, Neat1, Prss50, Rab37, 2410004N09Rik, Slc15a3, Ifit1, Slc24a1, Mc1r, Gm10509, Opn1mw, Cd164l2, Susd3, C3ar1, mt-Nd3, Kcna5, RP23-395C1.4, Ak7, Mx1, Dach1, Gjb6, 1500015O10Rik, Acer2, Serpinb8, H2-T23, Gpr64, Gm15821, RP24-199M24.1, Fgf7, Epha2, AF251705, Uxt, Six1, Gm8213, Slc6a3, RP23-400A19.3, Aldoart2, M1ap, Neurod4, Col5a3, Trpm1, Tmem215, Cd200r2, Irs4, Gm16505, Cyp3a13, Ptgs2, Thrsp, Rsad2, Zbp1, Kcnj14, Cnn2, Ptprc, Gm14680, Aoc3, Alox5ap, Syce1l, Adm, Ptpn3, Fam83g, 4932431P20Rik, Pcdhb6, Ncf1, Trpm6, Gm14340, Spock3, AI848285, Tnfrsf8, Gm13194, Rdh8, Ier3, B430306N03Rik, Gm13547, Olfr288, Arl4d, Evi2a, D330023K18Rik, Psmb8, Klk6, Sag, Ebf1, Scn4a, S100a6, Gm15491, Gabrd, Hoxa7, Hbb-bs, Palb2, 4732414G09Rik, Gm16973, 1500015A07Rik, Gm3095, Gpr84, Zic1, Plscr1, Tmem71, Pmp22, Gm14412, Gm14418, RP24-338G10.2, Cdh19, Anln, Ctss, H2-D1, C4b, Mylk3, Gbp9, Abcg3, Fabp12, Snhg5, Clec7a_12, Col4a4, mt-Nd4l, Ninj2, Cdhr3, Tcf24, Flnc, Xkr5, Mall, Best1, Serpine1, Ecscr, 3222401L13Rik, Gm14066, Tppp3, RP23-76P9.1, 4930447C04Rik, Mapk12, Bcl3, Mterf1b, Vim, Ly9, Pvrl4, Fbxw15, Gfap, Enpp2, Gm12907, Asic4, Zc3hav1, Ramp3, Saa3, Gpr179, Txlnb, Slc2a12, Fabp4, Treml2, Slc1a5, Tnfsf10, Rasl11a, RP23-298I7.4, Dct, RP24-285E3.2, Apol9b, 2410004P03Rik, Parp9, Gm14342, Gmfg, Oasl2, Gm9776, Eng, Slfn8, Prss54, RP24-155O13.4, Muc4, Gckr, Arhgap19, Slco4a1, Cd22, Cyba, Slco2a1, Armc4, Ttr, Lilrb4, Trpv4, Esrrb, Litaf, Pcdhb11, Fcrl1, Mboat1, Ifih1, Cirbp, Cdca5, Acy3, Ehhadh, Prdm13, Ccl3, Fzd10, Igtp, Cacna2d4, Tnf, Cpa4, Sp100,

	5930412G12Rik, Gm2830, A930033H14Rik, Rplp2-ps1, Iigp1, Adipoq, Samsn1, Pou4f1, Cyp1b1, Wnt9a, H2-K2, Iqca, 7630403G23Rik, Ppm1j, Hfe2, Asgr1, Parp10, 9330111N05Rik, Gh, Sp110, Polg2, Irgm2, Gbp2, Gm11738, Hck, Gjb1, Gm12326, Defb42, F2rl1, Tyms, 1700001O22Rik, Aspg, 9530026P05Rik, Rps18-ps3, Ccdc153, F830016B08Rik, Trim30a, Mgp, Nxnl1, Cited1, A830019P07Rik, Ptpn7, Lin28b, Ighm, Ighg2b, Rom1, Mmp12, Col6a1, Gm11944, Ip6k3, Trim30d, Pax2, Gm12183, Ppp1r14a, Fancf, Gnb3, Ddc, 2010001A14Rik, Hhex, Trh, Cfd, Il21r, Lsp1, Exoc3l4, Pomc, Col9a3, Il1a, Fkbp7, Gm12033, Asb14, Hpdl, Arl11, Bdkrb2, Krt9, RP23-8J15.3, BC061194, A330094K24Rik, C1qc, Igf2bp2, Gstm7, Agt, Gm15417, Ccl12, Mobp, Cd52, Ascl4, Steap1, Kazald1, Iyd, Tinag, Siglecg, Hbb-bt, Rpl27-ps3, Lyz1, Lyz2, A530053G22Rik, Bhlhe23, Pcdhb3, Rasa4, Rln3, Gpr152, Sox6os, Stap2, Hmox1, Gm15462, Parp14, Klhl40, Cldn2, Tnnt1, Six3os1, D730003I15Rik, Cdhr2, Cd180, Uba7, Gpr6, Ankrd33, Wnt9b, Pbld1, Tlr13, Clcnka, Cpa2, Krt7, Irx3, Scn7a, Tusc5, Tap1, Cd86, Meox1, Gstp2, Spag16, Spp1, Dmp1, Ggta1, Lgals9, Cela1, Gm15644, Oas1a, Fam19a3, Scgn, Pisd-ps2, Mfsd7a, Gm9767, Gm16233, Zfp825, Mslnl, Dhx58, Sh2d6, Pla2r1, Pycard, Phf11b, Rtbdn, Pon3, Gm11375, RP23-408A1.2, H2-Q4, Olr1, Scn4b, Fth1, Gchfr, Pcdhb8, RP24-79F8.3, Efemp1, Gm13939, Gm10136, Pdcd1, Barhl2, Fam46b, Mpp4, Tmem125, Phf21b, I830012O16Rik, Sult1a1, Fbln5, Naip5, Gm10244, Rnf125, Gm694, E230025N22Rik, Eya4, Trem2, Tgm2, Traf3ip3, Tnmd, Prss23, Retn, Cabp4, Slc25a5-ps, Lat2, Clic5, H2-Q6, Pou4f2, Plekhf1, Hif3a, Pnlip, Rs1, 4732491K20Rik, Mycbpap, Hdc, Art3, Plvap, Gm9918, Gabrr2, Crygc, Medag, Nkx2-9, Cuzd1, Apol6, Pik3r5, Sfi1, Plek, Gm13334, RP23-8J15.1, Gm3625, Cidec, Cpne9, Xdh, Tfpi, Gm15547, Pdxk-ps, Fam167b, Ly6c1, Ifi27l2a, Fev, F630040K05Rik, Cd14, Mab21l2, Cox8b, Cd68, 4931440L10Rik, Arhgap15, Tfap2d, Sptssb, Crybb3, Tmem243, Psg16, H2-K1, Cmah, Prl, Zfp185, Fgfr4, Havcr2, Unc13d, Xlr, RP23-405O11.1, Cd209d, Chrna6, Eif4ebp1, Prickle3, Cacna1f, Car3, Car13, BC033916, Nphs2, Gm12992, Gbp6, Defb1, Naip2, Crygd, Gm12300, Rnase4, Gm17673, 1700092M07Rik, Sec14l5, Arr3, Tuba8, Upk1b, Gng11, Selenbp1, Amy1, Mageb3, Slc25a45, Il33, Ms4a6d, Tnni1, Pkp1, Gm20033, Agmat, Tmem52, RP24-89C18.1, 9930004E17Rik, Itgb3, Gipr, Gm11639, Usp18, Pmaip1, Oasl1, Abca8a, Sostdc1, Mndal, Samd11, Tfap2b, Maff, C1qb, H2-Q7, 4933439C10Rik, Gbp3, Gm14114, Foxb1, Mfrp, Apoe, Gm17199, Myl2, Smok2b, Rspo1, Nxnl2, Cartpt, Rras, Haglr, Hcar2, Gngt2, Phpt1, Gm4951, Ube2l6, Mlph, Lactbl1, Lrrc36, Plin4, Card14, Rorc, Gbp7, Tcf15, Adamts19, Il2rg, Dnali1, Endou, Hbb-y, Alyref2, Pde6c, 4930547E08Rik, Pspn, Lbp, Crb1, Galnt15, Gm13135, Skint3, Isg15, Ptgir, Mip, Tcp10c, Slc36a2, Clec4e, Gm4924, Tfap4, Irgm1, B230206H07Rik, A230060F14Rik, Acvrl1, Crabp2, St14, Snx20, Vwf, Rgs9bp, Shisa3, Chrdl2, Tmem72, Mocos, Apod, Gm8093, Mb21d1, Ddx60, Anxa1, Fcgr2b, Serpinb1a, Rax, Gm9946, Cxcl12, Cryba4, Adssl1, 2600006K01Rik, Zc3h12a
**Ozone =341**	Vwa3a, Wfdc2, Col8a2, Gm5087, Man2c1os, Angptl2, Adamts12, Cdkn1c, Pcolce2, Gm9574, Gm5535, Fzd7, Htr2c, Gm16938, Tsga10ip, Atp4a, Slc14a2, Mlf1, Gm11425, AI607873, Tcea3, Cd59a, Gmnc, Defb11, Serpinb1b, Cdh3, Slco1a5, Prlr, Ebf3, Gm15853, Nfkbie, Tuba1c, Rragb, Prps1, Oit1, Gm16098, Cxcl10, Rrh, E230013L22Rik, Exo1, Pabpn1l, Col8a1, 4933416I08Rik, Cxcl16, Nid1, Nkx6-1, Slc26a9, Gm9922, AA414768, Pdgfd, Col4a3, Itih2, Pcolce, Hydin, Perp, Frrs1, RP23-285E19.4, Gpr1, Gm5913, Slc47a1, Pla2g5, Gja4, Spata31d1b, Rprd1b, Kcne2, Trp73, Gm20748, Tmem30a, Catip, Gm26622, Zic4, Klhdc1, Aspn, Wbscr27, Mx1, Scara5, Smarca5-ps, Gm10134, RP23-45M23.6, 1500015O10Rik, Slco1c1, Tekt3, Glp1r, RP23-18G1.2, Serping1, Abca4, Pgpep1l, Cldn9, Spata18, Best3, Baiap3, Irs4, Hcar1, Sash3, Kif9, Mettl21c, Pla2g2d, Ces1d, Magel2,

	B3gat2, Nupl2, Tgfbi, Dmrt3, Spint2, St6galnac2, Nedd1, Glra2, A730046J19Rik, Car12, Tle4, Tc2n, Siah1b, Col4a4, Trpm3, Adra2a, Gsc, Ppp1r36, Ccdc64b, Emp3, Ak9, Enpp2, Baiap2l1, Gm26682, Fgl2, Ubxn10, Lypd1, Dct, Slc25a35, Gabrq, Mycl, Gm853, A730017C20Rik, Alg5, Trpv4, Gm20430, Cd74, Nhlrc3, Lincenc1, Ifi203, Slc6a12, Col4a5, Uncx, Acy3, Krt8, Wdr96, Wdr77, Kl, 4930529M08Rik, Alox12, Ebf2, Gm26619, Fbxo22, Chrna3, Ccdc114, Fmo1, Parp10, Klra2, Gck, Vmn2r-ps3, Kng2, 1810019N24Rik, A230072C01Rik, Armc3, Cyp2f2, Cep76, Mgp, Rab20, Ighm, Glce, Msx1, Olfr574, 9330175E14Rik, Csrp2, Lhx1, Aatf, Sema3b, Was, Smoc1, Pear1, Col9a3, Itpa-ps2, Nek2, Tob2_11, Asb14, A530084C06Rik, Ccdc150, Adh1, Btc, Vat1l, Fsbp, Rcn3, Slc16a8, Mapk15, Gm15348, Atp12a, Tjp3, Scn5a, Zic3, Steap2, Steap1, Zscan10, Cd5, Corin, Efhc1, Lhx5, Wnt8b, Gm101, Rab19, Pltp, Cldn2, Mkrn2, Gspt2, Uba7, Fap, Nsun4, Npr1, Wdr52, Sybu, Tbx18, Naf1, Lrriq1, Ngb, Vkorc1l1, Tank, Tmem196, Gm10157, Vpreb1, Gm10220, Trav3-4, Igfbp2, Pon3, RP23-117O11.4, Ttll6, 1700007K13Rik, 5430416O09Rik, Ranbp3l, Etaa1os, Otx2os1, Krt2, Chac1, Nek5, Rassf9, Col6a3, Gm20621, Sod3, Pgam1-ps2, Arhgap36, Otx2, D6Ertd527e, Foxp2, Crb2, Myct1, Zic2, Acox2, Inmt, Cdh9, Wisp1, Igf2, Elovl7, Slc16a2, Svopl, Fam188b2, Gm5069, Tmem184a, Mid2, Mcm6, Syna, 4930486L24Rik, Tgds, Mab21l2, Cnga3, 1700026L06Rik, Fkrp, Gm13807, Slc16a9, Zfp185, Tcn2, Stap1, Hist1h2ae, Pax3, A4galt, Gbp6, Crygd, Gm609, Dynlt1b, Ccl7, RP23-69L13.3, Acot3, Lypd2, Ace, Col17a1, Col12a1, BC067074, Gpx8, Fst, Lox, Txndc2, Sostdc1, Zfp963, Oas1b, Tfap2b, Gm10762, Sulf1, Slc3a1, Trim5, Cartpt, Cab39l, Tmem255a, Rsph1, Slc37a1, Ube2l6, RP24-286H7.4, Nsun3, Adrb2, Mns1, Irx6, Zic5, Slc38a4, Lbp, Krt80, Sfrp5, Gm17315, Tspan10, Col18a1, Cngb3, Lepr, Ephx1, Fzd4, Gjb3, Gyltl1b, Cpn1, Katnbl1, Kcnj13, Pon1, Mlkl, Ooep, Arhgap1, Ubox5, Gm13330, Spon2, Itm2a
Intersection for all the 3 factors (n=17)	Wfdc2, Serpinb1b, Cdh3, Col8a1, Col4a3, Kcne2, 1500015O10Rik, Col4a4, Enpp2, Trpv4, Col9a3, Steap1, Cldn2, Pon3, Zfp185, Sostdc1
In both 17M and E4 (n=146)	Itgb4, Wfdc2, Slc4a5, Carns1, Nkx6-2, Fbxo32, Nt5e, Rdh5, Fign, Xaf1, Rhog, Wdr86, Cyp27a1, S1pr5, Rtp4, Serpinb1b, Cdh3, Ifi44, Avpr1b, Apol9a, Nlrp5-ps, Lhb, Col8a1, Dynlrb2, Folr1, Ifitm3, Irf7, Slfn9, Col4a3, Aqp1, 4930429F24Rik, Shox2, Lrrc23, Tmem212, Ifit3, Homer3, Kcne2, Apold1, Ifit1, Ak7, Dach1, Tmem215, Ptgs2, 1500015O10Rik, Serpinb8, Fgf7, Ptpn3, Evi2a, Klk6, Zic1, Pmp22, Gm14418, Anln, Col4a4, Cdhr3, Flnc, Tppp3, Vim, Enpp2, Zc3hav1, Slc2a12, Slc1a5, Rasl11a, Apol9b, 2410004P03Rik, Parp9, Oasl2, Iigp1, Armc4, Ttr, Trpv4, Litaf, Mboat1, Ehhadh, Igtp, Wnt9a, Iqca, Hfe2, Gh, Gjb1, Gm12326, Ccdc153, Trim30a, Trim30d, Ddc, Col9a3, Hpdl, Igf2bp2, Gstm7, Mobp, Steap1, Iyd, A530053G22Rik, Bhlhe23, Parp14, Cldn2, Tnnt1, Six3os1, Clcnka, Irx3, Spag16, Pon3, Tmem125, I830012O16Rik, Prss23, Pou4f2, Pnlip, Mycbpap, Hdc, Gabrr2, Nkx2-9, Ifi27l2a, Cox8b, Prl, Zfp185, Car13, Gng11, Tnni1, Usp18, Sostdc1, Gbp3, Mfrp, Lrrc36, Dnali1, Lbp, Isg15, Irgm1, Shisa3, Tmem72, Apod, Ddx60, Serpinb1a
In both E4 & O_3 (n=132)	Vwa3a, Wfdc2, Col8a2, Angptl2, Cdkn1c, Pcolce2, Fzd7, Htr2c, Atp4a, Slc14a2, Mlf1, Tcea3, Cd59a, Gmnc, Defb11, Serpinb1b, Cdh3, Slco1a5, Prlr, Ebf3, Tuba1c, Oit1, Rrh, E230013L22Rik, Col8a1, Pdgfd, Col4a3, Pcolce, Hydin, Perp, Pla2g5, Kcne2, Trp73, Catip, Zic4, 1500015O10Rik, Slco1c1, Tekt3, Glp1r, Serping1, Abca4, Cldn9, Spata18, Best3, Baiap3, Kif9, Magel2, Tgfbi, Dmrt3, Spint2, St6galnac2, Car12, Tc2n, Col4a4, Trpm3, Gsc, Ppp1r36, Ak9, Enpp2, Baiap2l1, Ubxn10, Gm853, Trpv4, Col4a5, Uncx, Krt8, Wdr96, Kl, 4930529M08Rik, Ebf2, Chrna3, Ccdc114, Armc3, Rab20, Msx1, Csrp2, Lhx1, Sema3b, Col9a3, Vat1l, Fsbp, Slc16a8, Mapk15, Tjp3, Scn5a, Zic3, Steap2, Steap1, Corin, Efhc1, Lhx5, Wnt8b, Gm101, Pltp, Cldn2, Fap, Npr1, Wdr52, Lrriq1, Vpreb1, Igfbp2, Pon3, Ttll6, 700007K13Rik,

	Otx2os1, Nek5, Rassf9, Col6a3, Sod3, Otx2, Foxp2, Crb2, Zic2, Acox2, Inmt, Igf2, Elovl7, Slc16a2, Tmem184a, 1700026L06Rik, Gm13807, Slc16a9, Zfp185, Tcn2, RP23-69L13.3, Ace, Col17a1, BC067074, Gpx8. Sostdc1, Sulf1, Cab39l, Rsph1, Mns1, Zic5, Lbp, Sfrp5, Col18a1, Lepr, Ephx1, Fzd4, Gyltl1b, Cpn1, Kcnj13, Pon1, Ooep
In both 17M & O_3 (n=33)	Wfdc2, Serpinb1b, Cdh3, Pabpn1l, Col8a1, Col4a3, Mgp, Kcne2, Mx1, 1500015O10Rik, Irs4, Col4a4, Enpp2, Dct, Trpv4, Acy3, Parp10, Ighm, Col9a3, Asb14, Lbp Steap1, Cldn2, Uba7, Pon3, Mab21l2, Zfp185, Gbp6, Crygd, Sostdc1, Tfap2b, Cartpt, Ube2l6
