# Supplementary material for: Age- and ApoE Genotype-Dependent Transcriptomic Responses to O3 in the Hippocampus of Mice
Source: Int J Mol Sci. 2025 Mar 7;26(6):2407. doi: 10.3390/ijms26062407 (PMC11942628; doi:10.3390/ijms26062407)
Supplement: Supplementary file 1 [file ijms-26-02407-s001.zip › Supplementary Table S2b List downregulated genes in the Venn diagram.pdf]

Supplementary Table S2b. List of downregulated genes of the three-factors (genotype, age and treatment) and the intersections in the Venn diagram

DEGs (Intersection)	Downregulated Genes
Genotype E4 (n=1011)	Chrd, C030037D09Rik, Bid, D030047H15Rik, Prss16, Abca12, Gm17597, N4bp2l1, Rpl28-ps1, Aim1l, Nnat, Gm3764, Cdc7, Hrh2, Mst1r, Gm15494_11, Gm14341, Map4k2, Tmem145, Gm5087, Spag5, Gm26852, Myo19, Gm15328, Stard9, Papl, Evpl, Rmst, Ccdc159, Gm15631, Pisd-ps1, Gm15333, Gm13056, Obscn, Zfp853, C630016N16Rik, Gm16845, Cplx3, Pparg, Gm4861, Phf2os1, Pabpn1, Limk1, G530011O06Rik, Gm14169, Nfu1, 9430015G10Rik, Fxyd6, Gm17168, A230083G16Rik, Kif1a, Pirt, Hes7, Csmd2, Rreb1, 4933427E13Rik, Gm13563, Tmem191c, Szt2, H2-M10.2, Pla2g3, Myom1, S1pr4, Mcmdc2, Cdc20b, Gm26541, 5031434O11Rik, Nlrp6, Trim72, Nr4a2, Tpm2, Gm14827, Plekha6, Skint1, Gm5415, RP24-540G19.3, Myo1a, Epha8, Slc16a3, Dgkq, Nphp3, 1700110I01Rik, Gm14198, Spata45, Tmem150a, Rnasel, Gm26549, Dmrt2, Nr6a1, Map2k6, Notum, Stx19, Myh7b, Gsg1l, Zbtb48, Tas1r1, Zmym6, Cep70, D930048N14Rik, Hook2, Mirg, Cygb, Pbx4, Wfs1, Itpr3, Pkn3, Pdss1, Cdk18, Gm12198, Col26a1, Tbx1, Tha1, Kif14, AW047730, F420014N23Rik, RP23-16F4.2, Gm16001, Arhgdig, Efcab12, Gdpd5, Rdh13, Gm12600, Pnpla3, Gm11974, 1110018N20Rik, 2900076A07Rik, Rgl2, Htr6, Slc9b2, Igsf9, Nrg4, Fezf2, Atxn7l2, Tmco5, 1190005I06Rik, Dcbld2, Fam131c, Tmsb10, Fam198a, Gm10010, Gm14270, Prr5, 9430037G07Rik, Aloxe3, Alox8, Cntnap5a, Doc2a, Cpsf4, Tmem132e, Esyt1, 0610009L18Rik, 1700071M16Rik, Gm15512, Robo3, Hpcal1, Rhbdf1, Ntn5, Gm10543, Camk2n1, Gm5111, 4931403E22Rik, 9230104M06Rik, Plk4, Gm13601, 1700020D05Rik, Pla2g4b, Gm12335, AI413582, Sh2b2, D930048G16Rik, Fam124a, Col7a1, Fgd2, E530001F21Rik, Camk2n2, Fam71e1, Efcab4b, Tex40, AI480526, Ddx26b, Tacr3, Fank1, Pik3cd, Asphd1, BB557941, Khdc1c, Atg16l2, Gm16701, Slc4a3, Pcyt2, Pcgf2, Lama3, 4930426I24Rik, 4921507L20Rik, Gm13883, Slc4a1, Lyrm9, Mapk13, Sync, RP23-321N22.11, Zkscan2, A330076H08Rik, Trmu, Wnt7b, Hnf1b, Pxdn, Hba-a2, Hba-a1, Kcnab3, Batf3, Optc, Gpr26, Prx, Serpini1, Peak1os, Plxnd1, Sirt4, Rps11-ps2, Gm13561, Pip5kl1, Col5a1, Kcnq4, Fgfr3, Piwil2, Ryr1, Fam98c, Gm14548, Gpsm1, Lhx3, Eqtn, E230016M11Rik, Nktr, Vipr1, Kifc5b, Gm12063, Tmem25, Gm16049, Atp8b2, Dffb, Prkab2, Cox11, Cyp11a1, 1700037H04Rik, Gm12503, Ap1m2, 9130024F11Rik, Gm16105, Myl3, Gm13341, Gm11732, Fat2, Gm9929, Myo1h, Cdc25b, Gm13493, Gm17111, Sbk3, BC048546, Ankrd24, C230004F18Rik, Tktl1, Plxna3, Map3k6, Sytl1, Tatdn3, Adamtsl5, Ccdc184, Gm16485, Pclo_11, Sec14l3, Plekhg4, Shd, Pmfbp1, Adamts16, Col16a1, Fbln2, Wsb1, RP24-87A4.1, Kdf1, Kcnip3, Olfm2, 2610005L07Rik, Gm20405, 1700023F06Rik, Slc39a2, Zfp473, Cwh43, Trank1, 1700030C14Rik, Plcxd2, Col4a2, Frmd3, Gm13830, A330102I10Rik, Gm26876, B430203G13Rik, Gm13485, Garnl3, Clasrp, Fxyd7, Snhg4, Fsd1, Hdx, Uggt2, Gm10805, Kcnt1, Pak6, Hrk, Filip1l, 4632411P08Rik, Myh8, Necab2, Nmbr, Ppm1m, Gm4793, 2900041M22Rik, Wnt5b, Jak3, Exoc3l2, Susd2, Gm16008, Gm12446, Plekha4, Wnt2, Gm20707, Podn, Echdc2, Lin7b, Inha, A630023P12Rik, Cplx1, Cox17, Gm15726, Mmel1, Gm13235, Cdh20, 4930563E22Rik, Tbc1d9b, Mcf2l, Evl, Hhipl1, Meg3, Gm26653, A830073O21Rik, 2610203C22Rik, Adck5, Ccnl2, Gm15518, Hbb-bs, Fxyd5, Cyp2j8, Dlk2, Gm9947, Tmem255b, Pagr1a, Acrbp, Hsd3b6, Grm4, Radil, Col24a1, Il1rapl2, Srrm4os, Gm15832, Racgap1, RP24-268C13.1, Gm17322, Gm21989, Zfp648, Sgcd, Ssh3,

	Obsl1, Grm2, Scnn1a, Rhbdl1, Gng13, Cd44, RP23-114N1.8, Gm12264, A730056A06Rik, Has1, Xndc1, Dynlt1a, Erdr1, Sema4g, Ninj2, Htr5a, Acp6, Arhgef15, Espn, Abcc5, Gm21811, Fhod3, Spata32, Alox12e, 4933427I22Rik, Gm13340, Fcho1, Gmip, Scd4, Gm11419, Sgpp2, Gpr25, Hook1, Ntng2, Ybx2, Csf2ra, 4930555F03Rik, Gm11870, Spaca6, Akap8l, Asl, Hsf4, Adamts2, Gm11492, Hexdc, Gnas, Fcer2a, Eml5, Sox7, A330023F24Rik, Gdpd2, Rbm3os, Dpysl4, Adam8, Cbln4, H2afj, Col28a1, Gm13029, Bzrap1, Prox2, Prrt2, C230037L18Rik, Usp28, D030046N08Rik, Tmsb15b1, Hist2h2bb, RP23-61B8.2, Plxdc1, Slc16a11, Tcte2, Pcsk2os2, RP23-285F13.1, Ypel4, Neil1, Tnfrsf18, Gm12930, Scn1b, Tmem40, Trat1, Syne4, Cyp46a1, Gm1043, Prm1, Alpk1, Cemip, Pah, Zfp692, 4930579G18Rik, Gm13882, Slc25a37, Frs3os, Gm14532, Gm13530, E430024P14Rik, Lman1l, Col23a1, Gm7393, BC030499, Zdhhc22, Nostrin, Gramd2, Gm12750, Gm11638, Gm26703, Ccdc84, Mup5, Nudt8, Zmiz1os1, RP23-291B1.1, 2810055G20Rik, Rasal3, Ica1, Rpl35, Apaf1, Apol7d, Lrrc16b, Gm4779, Zfr2, Gm2830, Celsr3, Sla2, Necab1, Gm11434, Lmnb2, Trpm4, Kif17, Gm2449, A230050P20Rik, Ulk3, Hrh1, Nkx6-3, Lrrc39, Rnf223, Arntl2, Clgn, Gm10390, A230052G05Rik, Gm26724, Msc, Gsta3, Pcsk5, Gm12963, Fut9, Ccdc176, Tdrd5, Ldhd, Coro2a, Gm7854, Gm14422, Zfp541, Gm26734, Slc6a7, Ost4, Rap1gap2, Wnt5a, Kcnj9, Exd1, Lsm8, Gm4875, Gm15411, H2-M6-ps, Prdm9, Gm3985, Serpinb5, Ly6g6e, Galr2, Banp, Pcsk1n, Psrc1, 9430041J12Rik_11, Ighg2c, Sypl2, Mios, R3hdm4, Gm15594, Lhx2, Zfp57, RP23-48C24.3, Tspan17, Edn1, Entpd2, Hdac7, Vdr, Leng8, Gm15247, Gm7870, RP23-189P1.2, Smpd4, Trpm2, Rps6kb2, Gm15416, Tmem181b-ps, Gm17116, Tmem44, Atg9b, Qk_12, Chrna4, Il1b, Greb1l, Ccdc88b, Zfp341, A930011O12Rik, Abcc8, Otof, Spsb3, A230006K03Rik, Adamts10, Pola2, Itgal, Grin1os, Kcnmb4os2, Khdc3, Tecta, Gm26761, Prkd2, Otop2, Sfxn3, Proscos, RP23-188J8.2, 5730522E02Rik, Rxfp1, Adcy4, Ripk3, Kcnh4, Ushbp1, Rell2, Muc19, Kcnh6, Cav2, Lipm, Gpr111, Gm12197, Gm16892, Dennd3, Serac1, Spocd1, Fam83b, Sh2d3c, Clcn2, Thpo, Ror2, Hbb-bt, Adam33, Vmn2r84, Wdfy1, Gm10557, 1700034I23Rik, Gm9804, Fam83h, Gm16314, H2-Q10, Gm27247, Cdh23, Rnasek, Calca, Calcb, Stap2, RP24-548K2.3, Rpa3, Sox17, Sstr1, Abca17, Pnisr, Gm16355, Ltk, Gm15535, Fam136b-ps, Ppp1r3g, Gm27000, Rpl31-ps16, Ghrl, Asprv1, Gm10080, Gm4211, 4933408B17Rik, Ccdc183, Pon2, Mst1, Gm15317, 9830144P21Rik, 5730405O15Rik, Gm12447, Gm26782, Gm26783, Gm16630, Btla, Rufy2, Trim75, Col5a2, Tmem202, Ube2cbp, Gm26786, Ildr1, Fbxo2, Zgpat, Gm15492, Rspo2, Gm26792, Atp10a, RP23-326F22.4, Tia1, Gm13391, Donson, Dmp1, Rgsl1, Dcdc2b, Kiss1r, Mei1, Bola2, Ebf4, Izumo4, Cmc2, Hes5, Nadsyn1, Pisd-ps2, Lin37, L3mbtl1, Fam193b, Pdlim7, Cntnap3, Resp18, Apoa2, Ftx, Atcayos, Vwa5b2, Cml5, 6530402F18Rik, Gm15574, Agrn, Myh4, 5830411N06Rik, Pappa2, Gm7276, Vwce, Sncb, Pth1r, Gm14164, RP23-158A22.1, Gm15826, Gm26797, Sptbn4, Gm9752, Atpif1, A930005H10Rik, Trmt13, Cacna1g, Alkbh6, Helq, Rpl23a-ps2, Cyp4f18, Ube4bos3, Tlr5, Gm7353, Ctgf, Mtfr2, Rab4b, AI854703, Ccdc124, Gm9989, Gm17195, A830031A19Rik, Amn, 1110008P14Rik, Ankrd9, 4930539E08Rik, RP23-392G15.1, Rhov, Lemd1, Clec2l, Gm16047, Gm26809, Panct2, A230087F16Rik, Gm26819, Hsd3b1, Nox1, Tle6, Cenpm, Ephx4, Ulk2, Clk4, Gm16586, 5830416P10Rik, Gtpbp2, Gm27016, 5830462I19Rik, BC068157, Dleu7, Slc23a3, H2-Bl, Dgcr6, Arrdc1, Ncapg2, Ttc9b, Hsf2bp, RP23-48C24.2, Klhl17, Vmn2r85, Slc22a7, Ddx4, Kif5a, Hkdc1, Tacr2, Mmp21, Emx1, Atp11b, C2cd4c, Gm13375, Dsc3, Ptp4a3, Tsks, Ccdc142, Fam109a, Serpina12, Gm7061, Dctd, Cntn5, Nfkbid, Gm12404, Gm12246, Hbq1a, Arhgef1, Dennd6b, Gm12576, A330009N23Rik, Gm26839, Acat3, Pradc1, Dna2, Snhg11, Rnaseh2c, 4931422A03Rik, AU022754, Prkag2os1,

	Adamts17, Uba52, 9530036O11Rik, Clnk, Ccdc136, Ovgp1, Amdhd1, Gm15496, Gm13589, Gm26843, Eno3, Gm26848, Lgi2, Vstm2b, Ccl27a, Pcsk2os1, Myh2, 9430021M05Rik, Slc27a2, Rgs11, Prrt4, Tmem243, Gm12069, Nsun5, Psg16, 3632454L22Rik, Lhfpl5, Bglap3, 5830428H23Rik, Sec1, Nckap5los, Capn12, AW495222, Zcchc7, Hcn3, Card10, Gm14556, Gm12473, Stxbp2, Gm10742, Gm13716, Ak6, Gli1, Serp2, Lefty2, Gm6756, Coro6, Pde4c, Snx22, Ppl, Shkbp1, Esr2, Amy1, A530058N18Rik_12, Tnnt2, Rgag1, Gm27032, Fam220-ps, Kcnn1, 9330185C12Rik, Il17d, Aifm3, Ap1g2, Cyp2s1, D430041D05Rik, Crip3, Gm15494_12, Crhr1, Shc2, Gm6537, Fancd2, Adcy7, Egfl7, Spin2c, Lyzl4, Tcea2, Rnf207, D830026I12Rik, RP23-184B11.7, Papln, Gm17201, Srrm4, Ccdc129, Ercc2, Neil2, Matk, Rasal1, Engase, Gm14257, Igsf21, Gm12539, 1700037C18Rik, Usp35, Dok3, Lad1, Cables2, Gm8584, Rac3, RP24-287C2.2, Gng10, Samd10, Bmper, Naa10, Casp1, Epha10, Apol7a, Snhg7, Pitpnm3, 2310026I22Rik, Ankrd16, Sbsn, Gpr150, Rian, Brd9, Gngt2, Gm8104, Gm20109, Adrbk2, Bcl2l15, Miat, 5730420D15Rik, Gm11841, Ces1c, Gm16022, E030019B06Rik, BC005561, Mapk11, Gm14507, Kcnc4, Psmb10, Ctrl, Gm10343, Chd3os, Gm5577, Nxph4, Uqcc2, Apol8, Treml4, Gm14830, Gm20421, Cd46, Cgref1, Gm6483, 9630013K17Rik, Cecr5, Zfp612, Aldh1a3, Pcdh15, Galnt15, RP23-52N17.1, Otc, Gm7568, Sema4a, Nbeal2, Gm26964, Gm11490, Lrrc45, Grb7, Camk2d, Gm26902, Rgl3, Avil, Snapc4, Pkib, Gm17112, Col19a1, Vill, Flt3, Krba1, Cyp4f15, Ccdc57, Gm10644, Gm12276, Cdc25c, Necab3, Prss8, Gm13754, 5430400D12Rik, Tmem30c, Npc1l1, Tsga13, Gm8582, Abcc2, Gm13205, C030005K06Rik, Spata19, 2810029C07Rik, Arpp21, Ttc14, Gm14137, Mien1, Gm2694, Vip, Gm15706, Hypk, Kifc2, Polq, Mroh8, Gm12359, Gm15445, Anks6, 4933405L10Rik, Enkd1, Kif22, 1700008F21Rik, Gm16039, Tle2
Age 17M (n=383)	Gm17249, Gm15342, Apex2, Nckap5, Sp8, Parm1, Atp1a4, Gm26854, Gm15591, Hcn1, Sox11, Fbn2, Mical3_11, Gm13134, Pirt, Tenm4, Kcna3, Birc7, Slc8a1, Gm6467, Morc2b, Slc14a2, Gm26536, Gm11201, Tnc, Ace3, Kcnq1ot1, Nr4a2, Cbfa2t3, Slc5a3, CH25-211A23.1, Atp2b3, Gm26592, Gm12108, Gm26546, Tbck, Parp16, Gm15240, Ldha-ps2, Gm11659, RP23-238L21.5, Lrrn4, C230038L03Rik, Gm15566, Fbxl12os, Gm9934, Brwd3, Ndst4, Gm15237, RP24-240G4.2, Gm4660, Map3k19, RP23-116J1.1, Sntb1, Capns2, Cbl, Gm15828, Gm26586, Gm10564, Adnp, Gm14489, Gm15957, 4930404H24Rik, AW551984, Lrrc7, Gm5801, Hs6st3, Gm19426, Fam135b, Nfya, Gm7347, Speer4a, 4933423P22Rik, Ptpru, Frrs1, Hs6st2, Gm14584, Gm15565, Gm20699, Myo16, Tmem91, Myo3b, Gm11914, Gm9816, Dnm3os, Atp6v1e2, Prss48, Tmprss15, Gm12816, Xylt1, Sox4, 4930578C19Rik, Gpr165, Htr1b, BC039771, Met, Ccdc8, C230004F18Rik, Gm11365, Syt17, Slco1b2, Col14a1, Gm12926, Gm16019, Gm10912, Bcas3os1, Xkr4, RP23-347F2.3, Gm16159, Gm12296, Tnr, Sfmbt2, Gm10369, Cyb561, Xirp1, Myh11, Cd55, Nos2, Gm26962, Gm26638, Gm16243, Nmbr, Gm11960, 2310075C17Rik, Fat3, RP23-400F13.1, Podn, Gpr82, Gm11969, Gm26648, 2610203C22Rik, Gm5878, Gm9947, B830012L14Rik, Mkln1os, Cndp1, Syne2, Chrna5, Rbm4, Htr1a, Mdga1, Bnipl, Gpr17, Gm13861, Gm26538, RP23-245G9.1, Ttc30a2, Slc15a5, Gm12122, Marcksl1, Trdj2, Fbxo47, Adamts2, 5031415H12Rik, 2010300F17Rik, Mmp15, Gm15807, Gm2974, C130026L21Rik, Gm13827, Zbbx, Kcng1, Zbtb40, Spink4, Gm17477, Gm12230, Gm15461, 4930517E11Rik, 6030443J06Rik, Gm15956, Gm2044, Gm20616, Tmem200a, Cdr1, Gm15606, Ube2t, Kif27, Slc16a14, Gm17494, Gm8546, Gm19967, 4930417H01Rik, 9430025C20Rik, Gm15742, Pcsk5, Gm11670, C630028M04Rik, Ccdc176, Tenm1, Tmem170, Gm14853, Tm6sf2, 1810026B05Rik, Gm11447, Gm6588, Myo18b, Gm11282, Gm21844, Aspdh, Gm16347, Trpc4, Esrp2, RP23-356A24.1, 5730422E09Rik, Gm26833, Elf5,

	Rsl1, Gm15520, Hmcn1, Gm16239, Gm6263, Igsf9b, Gm16316, Slc22a21, A630075F10Rik, Hspa5, Gm27209, Gm7887, Gm14174, Gm4810, Gm12064, Scn5a, Zdbf2, Zic3, Zdhhc23, Gm17828, Nov, Ccdc34os, Gm14329, 3110067C02Rik, Mc5r, Gemin2, Gm17473, Gm26779, Grin3a, Selenbp2, Cgn, Gm27253, RP23-190F21.2, Gm10544, 9530034E10Rik, Npbwr1, Gm26785, Slc27a3, Tex30, Grip1, Itgb2l, Gm5784, Gm9913, Sost, 4933413L06Rik, Serinc2, Nts, Zfp369, Gm14288, Gm14502, Gm14735, Hist1h4k, Krtcap3, Pdia4, Plk-ps1, Irs1, Gm15577, Map3k7cl, Gm15521, Gprin3, Cadps2, 4930445B16Rik, Cacna1g, Olfr691, Gm12136, Rab38, Galntl6_11, Gm26804, Ccdc151, Gm26807, Ccdc88c, RP24-86C3.1, 9630013D21Rik, Gm13736, Tomm6os, Zfp964, Tex38, Gm15764, Ppp3r2, Gm13783, Dmrta2, Nrg3os, Ror1, B130055M24Rik, Gm15673, Klhl1, Rps11-ps1, Gm15666, Gm15425, Gm27019, Gm26835, Gm13248, Tenm3, Oc90, Wdr95, Gm7452, Gm9982, Cdkl4, Gm9817, Gm15046, Anks4b, Gm26841, Gm10874, Nynrin, Gm21885, Gm12315, Mettl21b, Cntnap5b, Gm26849, Odf3, Klk12, Gm6341, Epha6, Sstr4, RP24-212L9.1, 2810407A14Rik, Ccdc81, Gm14556, Cenpf, Atxn7l1os2, Frmpd3, Gm16147, Mettl11b, Pgpep1, Ccdc60, Mybl2, Gm10561, Kcnn3, Crip3, Serpinh1, Hist1h2ad, Fras1, Npr3, Prdm12, Gm15397, Traf4, Gm11915, Etv1, Gpr161, Gm12141, Asxl3, RP24-217M7.4, Gm13965, Nell1os, Gapdhs, Figf, 4930447A16Rik, Pde11a, E030018B13Rik, Gm17709, Drd5, RP23-386K20.3, Gm3943, Slc18a1, 4930448N21Rik, 4931429I11Rik, Ppp2r2cos, Pcdh12, Gm16023, C1qtnf1, Pitpnm2os1, Arg2, Gpx2-ps1, Gm11490, Tmem74, Gm26904, Gm15732, Gm15609, Zfp872, Angptl3, Zim1, Gm17227, Gm15082, Mitf, Gpr139, Gm2245, Pou5f2
Treatment; Ozone (n=207)	Gm10030, Has3, Zfp382, Gm26854, A930029G22Rik, 6430590A07Rik, Boll, Cd177, 2610042L04Rik, Gm26537, Lcn2, Rpl35a-ps7, Ceacam16, RP23-99I11.2, Fam163a, Adora3_11, Rimkla, Gm12798, Eif5b, Fam228b, Gm10243, 2610020F03Rik, Blzf1, Vav2, Gm12717, Ptcd1, Gfm2, Thap2, A930018M24Rik, Pptc7, Gm10564, Gnb1l, Tnfrsf11a, 1200014J11Rik, Gm15479, 5330439K02Rik, Cdr2l, Tpmt, Gm12394, Gm17354, Ptpn1, Tnni3k, Rarres1, Gm15651, Gm14057, Gm2000, Gm27206, Msantd1, 6030445D17Rik, Arid5a, 2700029M09Rik, Tyk2, Rhot2, Pnpla7, Toe1, Gm12454, Gm27197, Creb3l3, Ccl9, Muc6, Gm16315, Fam186b, Rhoq, Ncf1, Cep112os2, Gm12353, 4930412M03Rik, Gm14296, Gm14172, Gm26656, Gm17108, Lrrc26, Pygm, Zfp319_11, RP23-191A12.6, Gm14066, Gm20111, Gm13630, Slc9a3, RP24-247B20.1, 1700003M07Rik, Gm26520, Gm26681, Gm15675, Gm12971, Gm10604, Gm21817, Gm15155, Rpl13-ps1, Gm11837, Gm17477, Plet1, D730045A05Rik, Enpep, Fam20a, Itpr1, Fam64a, Bcl2l10, Eif2d, 2810055G20Rik, Gm21028, RP23-2M16.2, Gm12485, AW011738, Paqr8, Ttc7, Nans, Sdccag8, Tmem8c, A830019P07Rik, Gm10705, Dlx1as, Rbm14, Gm17135, Atoh7, Oaf, Pomc, Hspb3, Gm13689, Gm11459, Gm11955, Reep4, Gm15774, A430104N18Rik, Gm4787, Gm14033, Gm12248, Zfp524, Lmf1, Ccdc34os, Rnf166, Ltb, Gpam, Gm13416, RP24-421J9.3, Klk11, Trim75, Krt7, Gm7541, Dmp1, Rps6-ps3, Pcbd2, Vmn1r90_12, Gm11725, Gprin3, Arhgap11a, Gm15835, Limch1, Sap30, Gm12468, Gm26816, Eme2, Cdh17, Ern2, Dio2, Ptprv, Gm8430, Gm15816, Gm14445, Foxp3, Rhd, 4732419C18Rik, Abi3, Gm15809, Zscan20, 1810010K12Rik, Gm7628, Txlna, Sytl5, Axin1, 4933427E11Rik, Nefh, Proser2, Sstr4, Gm17669, Atxn7l1os2, Mettl11b, Lhcgr, Gm13062, Matn4, Pcdhb14, Zfp276, Rasl10b, Pias4, Gm26875, Gm26881, Gm8540, Gm18048, 1700125G02Rik, Atp2a1, Gm11646, Gm9754, 4930448N21Rik, Adamts19, 4930518I15Rik, Gm16128, Gm13326, Gm10015, Gm12517, Gm26910, Rhox5, Rbm15, 4833417C18Rik, Oxt, Abcb4, Cmya5, Cyp2r1
Intersection for all the	

3 factors (n=0)	
In both 17M and E4 (n=3)	Pirt, Nr4a2, Nmbr, Podn, Adamts2, Pcsk5, Ccdc176, Cacna1g, 2610203C22Rik, Gm9947, Gm14556, Crip3, Gm11490, C230004F18Rik
In both E4 & O_3 (n=14)	2810055G20Rik, Trim75, Dmp1
In both 17M & O_3 (n=9)	Ccdc34os, Gprin3, Sstr4, Atxn7l1os2, Mettl11b, Gm26854, Gm10564, Gm17477, 4930448N21Rik
