# Supplementary material for: Age- and ApoE Genotype-Dependent Transcriptomic Responses to O3 in the Hippocampus of Mice
Source: Int J Mol Sci. 2025 Mar 7;26(6):2407. doi: 10.3390/ijms26062407 (PMC11942628; doi:10.3390/ijms26062407)
Supplement: Supplementary file 1 [file ijms-26-02407-s001.zip › Supplementary Table S3 DEGs E4 vs. E3 17M O3.pdf]

**Supplementary Table S3. Differentially expressed genes (DEGs) between E4 vs. E3 (17M) mice exposed to ozone**

| | Upregulated DEGs E4 vs. E3 (17M $O_3$) | | | | Downregulated DEGs E4 vs. E3 (17M $O_3$) | | |
|---|---|---|---|---|---|---|---|
| | **Gene Name** | **log2FoldChange** | ***p* value** | | **Gene Name** | **log2FoldChange** | ***p* value** |
| 1 | Hnrnpk | 0.28 | 0.039 | 1 | Gm47283 | -8.53 | 4.1E-11 |
| 2 | Gaa | 0.28 | 0.039 | 2 | Gm21887 | -7.31 | 1.2E-07 |
| 3 | Large1 | 0.30 | 0.048 | 3 | Gm20186 | -5.45 | 2.5E-06 |
| 4 | Eps15 | 0.31 | 0.048 | 4 | Gm11732 | -5.37 | 0.001 |
| 5 | Ppp2r2a | 0.31 | 0.036 | 5 | Cyp2b19 | -5.32 | 0.001 |
| 6 | Wasl | 0.32 | 0.041 | 6 | Gm24497 | -4.97 | 0.007 |
| 7 | Mast4 | 0.33 | 0.047 | 7 | Csn3 | -4.95 | 0.038 |
| 8 | Ndfip2 | 0.34 | 0.040 | 8 | Hba-a2 | -4.74 | 3.0E-19 |
| 9 | Actr2 | 0.34 | 0.040 | 9 | Myl3 | -4.73 | 0.010 |
| 10 | Ywhaq | 0.35 | 0.043 | 10 | 4930474M22Rik | -4.70 | 0.018 |
| 11 | Secisbp2l | 0.35 | 3.15E-02 | 11 | Umod | -4.67 | 0.028 |
| 12 | Ptp4a1 | 0.35 | 0.036 | 12 | Kap | -4.57 | 0.036 |
| 13 | Dipk1a | 0.36 | 0.043 | 13 | Ighg2c | -4.53 | 0.004 |
| 14 | Ndrg1 | 0.36 | 0.045 | 14 | Hba-a1 | -4.42 | 9.3E-37 |
| 15 | Map6d1 | 0.36 | 0.042 | 15 | 1700008A23Rik | -4.38 | 0.025 |
| 16 | Cacnb2 | 0.37 | 0.037 | 16 | Gm49709 | -4.29 | 0.041 |
| 17 | Gnai1 | 0.37 | 0.019 | 17 | Esr2 | -4.28 | 0.008 |
| 18 | Degs1 | 0.38 | 0.039 | 18 | Gm45219 | -4.25 | 0.041 |
| 19 | Slc24a3 | 0.38 | 0.034 | 19 | Hbb-bt | -4.22 | 2.5E-19 |
| 20 | Ctnna1 | 0.39 | 0.016 | 20 | Hbb-bs | -4.18 | 6.7E-31 |
| 21 | Frmpd4 | 0.39 | 0.047 | 21 | Tyrp1 | -4.12 | 0.015 |
| 22 | Npr3 | 0.39 | 0.049 | 22 | Gm44430 | -4.09 | 0.018 |
| 23 | Brinp1 | 0.39 | 0.044 | 23 | Gm42655 | -4.04 | 0.017 |
| 24 | Atp2b1 | 0.39 | 0.038 | 24 | Gm12089 | -3.80 | 0.034 |
| 25 | Inf2 | 0.39 | 0.016 | 25 | Gm47328 | -3.76 | 0.031 |
| 26 | Mtus1 | 0.39 | 0.043 | 26 | Gm10129 | -3.68 | 0.028 |
| 27 | Furin | 0.40 | 0.025 | 27 | Dnase1l3 | -3.65 | 0.038 |
| 28 | Slc24a5 | 0.40 | 0.035 | 28 | Mip | -3.53 | 0.001 |
| 29 | Tapbp | 0.40 | 0.018 | 29 | S1pr4 | -3.46 | 0.006 |
| 30 | Gsn | 0.41 | 0.040 | 30 | Cryba2 | -3.41 | 0.000 |
| 31 | C4b | 0.41 | 0.030 | 31 | Gm5087 | -3.37 | 0.023 |
| 32 | Myg1 | 0.41 | 0.049 | 32 | Fat2 | -3.20 | 0.001 |
| 33 | Rela | 0.41 | 0.046 | 33 | Cidec | -3.16 | 0.001 |
| 34 | Tfrc | 0.42 | 0.004 | 34 | Gm27002 | -2.98 | 0.031 |
| 35 | Lrrc8d | 0.42 | 0.034 | 35 | Prss8 | -2.93 | 0.001 |
| 36 | Rbm3 | 0.42 | 0.018 | 36 | Gm42133 | -2.88 | 0.031 |
| 37 | Scyl3 | 0.42 | 0.037 | 37 | Lrit3 | -2.86 | 0.016 |
| 38 | Wls | 0.43 | 0.030 | 38 | Gm15948 | -2.85 | 0.029 |
| 39 | Plxnb3 | 0.43 | 0.038 | 39 | Myo1a | -2.83 | 0.021 |
| 40 | Serinc5 | 0.43 | 0.010 | 40 | Gm36283 | -2.66 | 0.026 |
| 41 | Lamp2 | 0.43 | 0.034 | 41 | Gm29438 | -2.64 | 0.012 |
| 42 | Ankrd28 | 0.43 | 0.009 | 42 | Cryba1 | -2.59 | 0.023 |
| 43 | Gad2 | 0.44 | 0.030 | 43 | Gm43844 | -2.58 | 0.008 |
| 44 | Spock1 | 0.44 | 0.047 | 44 | Gm25596 | -2.55 | 0.010 |
| 45 | Zeb2 | 0.44 | 0.006 | 45 | H2-Q2 | -2.42 | 0.027 |
| 46 | Trim24 | 0.44 | 0.024 | 46 | Bc1 | -2.42 | 5.6E-09 |
| 47 | Cntnap2 | 0.44 | 0.013 | 47 | Lif | -2.42 | 0.012 |

| | | | | | | | |
|---|---|---|---|---|---|---|---|
| 48 | Rtl8a | 0.44 | 0.024 | 48 | Vwce | -2.40 | 0.049 |
| 49 | Abcc1 | 0.44 | 0.044 | 49 | Dsc3 | -2.38 | 1.2E-08 |
| 50 | Plekhb1 | 0.45 | 0.023 | 50 | 4930483P17Rik | -2.36 | 0.033 |
| 51 | Prps2 | 0.45 | 0.038 | 51 | Nhlh1 | -2.34 | 0.037 |
| 52 | Rnf13 | 0.45 | 0.006 | 52 | Adipoq | -2.32 | 0.014 |
| 53 | Scg2 | 0.46 | 0.017 | 53 | Gm36210 | -2.23 | 0.002 |
| 54 | Acsl1 | 0.46 | 0.009 | 54 | Mir212 | -2.22 | 0.012 |
| 55 | Wasf1 | 0.46 | 0.021 | 55 | Cryba4 | -2.20 | 0.002 |
| 56 | Gatm | 0.46 | 0.020 | 56 | Gja8 | -2.11 | 0.048 |
| 57 | Gm42413 | 0.46 | 0.027 | 57 | Gm36251 | -2.07 | 0.014 |
| 58 | Clic4 | 0.47 | 0.034 | 58 | D430020J02Rik | -2.07 | 0.002 |
| 59 | F420014N23Rik | 0.47 | 0.045 | 59 | Gm12446 | -2.00 | 0.034 |
| 60 | Lrrn2 | 0.47 | 0.036 | 60 | Gm37593 | -1.99 | 0.012 |
| 61 | Cul4b | 0.47 | 0.035 | 61 | Gm36529 | -1.99 | 0.032 |
| 62 | Fam234a | 0.48 | 0.048 | 62 | Sec14l3 | -1.96 | 2.5E-05 |
| 63 | Gm44103 | 0.48 | 0.046 | 63 | Pth2r | -1.91 | 0.005 |
| 64 | Slc32a1 | 0.48 | 0.040 | 64 | Mir341 | -1.90 | 0.021 |
| 65 | Aldh1a1 | 0.48 | 0.008 | 65 | Wdfy1 | -1.78 | 7.6E-21 |
| 66 | Stk39 | 0.48 | 0.023 | 66 | Sytl1 | -1.78 | 0.017 |
| 67 | Cacng8 | 0.48 | 0.029 | 67 | Gm25047 | -1.76 | 0.038 |
| 68 | Ddr1 | 0.48 | 0.046 | 68 | Trhr | -1.76 | 0.001 |
| 69 | Lpar1 | 0.49 | 0.023 | 69 | Gm7331 | -1.76 | 0.037 |
| 70 | Prr18 | 0.49 | 0.033 | 70 | Gm37266 | -1.73 | 0.034 |
| 71 | Adamts4 | 0.49 | 0.020 | 71 | Ly6g6e | -1.72 | 0.003 |
| 72 | Hapln4 | 0.49 | 0.042 | 72 | 9430085M18Rik | -1.71 | 0.011 |
| 73 | Cpt1a | 0.50 | 0.011 | 73 | Alas2 | -1.68 | 0.002 |
| 74 | Vamp3 | 0.50 | 0.023 | 74 | Calca | -1.66 | 0.019 |
| 75 | Ly6e | 0.50 | 0.043 | 75 | Dio3 | -1.66 | 0.014 |
| 76 | Mt2 | 0.50 | 0.029 | 76 | Dmrt2 | -1.65 | 0.029 |
| 77 | Mbp | 0.50 | 0.041 | 77 | Prkag2os1 | -1.60 | 0.040 |
| 78 | Plekhg3 | 0.51 | 0.026 | 78 | Cbln4 | -1.59 | 4.7E-08 |
| 79 | Thnsl1 | 0.51 | 0.035 | 79 | Fermt1 | -1.58 | 0.001 |
| 80 | Gabra5 | 0.51 | 0.037 | 80 | Trpm1 | -1.57 | 0.044 |
| 81 | Pcp4l1 | 0.52 | 0.043 | 81 | Tbx1 | -1.55 | 0.049 |
| 82 | Galnt17 | 0.52 | 0.045 | 82 | Lad1 | -1.53 | 0.002 |
| 83 | Pde8a | 0.52 | 0.011 | 83 | Gm6934 | -1.52 | 0.034 |
| 84 | Rgs3 | 0.52 | 0.028 | 84 | Casp1 | -1.51 | 0.001 |
| 85 | Fgfr2 | 0.52 | 0.010 | 85 | Prph | -1.50 | 0.016 |
| 86 | Tfeb | 0.52 | 0.047 | 86 | Krt2 | -1.49 | 0.018 |
| 87 | Fbxo32 | 0.53 | 0.011 | 87 | A2ml1 | -1.48 | 1.6E-05 |
| 88 | Ttyh2 | 0.53 | 0.013 | 88 | Gsg1l | -1.48 | 0.009 |
| 89 | Txnip | 0.53 | 0.038 | 89 | Gm45095 | -1.47 | 0.027 |
| 90 | Gjc3 | 0.53 | 0.004 | 90 | AU022754 | -1.45 | 0.004 |
| 91 | Pls1 | 0.53 | 0.045 | 91 | Vill | -1.40 | 0.003 |
| 92 | Itgb4 | 0.54 | 0.036 | 92 | Tnnt2 | -1.40 | 0.003 |
| 93 | Kirrel2 | 0.54 | 0.039 | 93 | Nr4a2 | -1.38 | 0.012 |
| 94 | Myrf | 0.54 | 0.019 | 94 | Dcn | -1.36 | 0.048 |
| 95 | Klf3 | 0.54 | 0.004 | 95 | Gm5242 | -1.36 | 0.031 |
| 96 | Ugt8a | 0.54 | 0.015 | 96 | Otof | -1.36 | 0.001 |
| 97 | Ermn | 0.54 | 0.019 | 97 | Gm15494 | -1.34 | 0.048 |
| 98 | Nr3c2 | 0.54 | 0.014 | 98 | Ahnak2 | -1.33 | 0.042 |
| 99 | Slco1a4 | 0.54 | 0.026 | 99 | Gm4131 | -1.31 | 0.045 |
| 100 | Slc12a4 | 0.55 | 0.030 | 100 | Wfs1 | -1.27 | 0.024 |

| | | | | | | | |
|---|---|---|---|---|---|---|---|
| 101 | Sema6a | 0.55 | 0.010 | 101 | Has1 | -1.27 | 0.047 |
| 102 | Bcas1 | 0.55 | 0.017 | 102 | Gm10605 | -1.25 | 0.007 |
| 103 | Kcnt2 | 0.55 | 0.030 | 103 | Grm2 | -1.24 | 5.9E-07 |
| 104 | Myo1d | 0.55 | 0.020 | 104 | Crybb3 | -1.23 | 0.018 |
| 105 | Sh3d19 | 0.55 | 0.023 | 105 | Tacr3 | -1.23 | 0.029 |
| 106 | Nr4a3 | 0.55 | 0.042 | 106 | Dctd | -1.22 | 0.003 |
| 107 | Mobp | 0.56 | 0.022 | 107 | Gpr101 | -1.21 | 0.002 |
| 108 | Gm17035 | 0.56 | 0.006 | 108 | Galnt15 | -1.20 | 0.002 |
| 109 | Susd6 | 0.56 | 0.016 | 109 | Amn | -1.20 | 0.009 |
| 110 | Plcl1 | 0.56 | 0.009 | 110 | Htra4 | -1.20 | 0.026 |
| 111 | Vim | 0.56 | 0.048 | 111 | Plxnd1 | -1.19 | 0.041 |
| 112 | Ccdc24 | 0.56 | 0.037 | 112 | Zfp648 | -1.18 | 0.026 |
| 113 | Mcam | 0.56 | 0.032 | 113 | Nostrin | -1.17 | 0.028 |
| 114 | Syt12 | 0.56 | 0.012 | 114 | Ghrl | -1.15 | 0.046 |
| 115 | Tspan2 | 0.56 | 0.004 | 115 | Cd44 | -1.14 | 0.008 |
| 116 | Kcnh1 | 0.56 | 0.032 | 116 | Gm8066 | -1.13 | 0.014 |
| 117 | Mal | 0.57 | 0.018 | 117 | Cox6a2 | -1.11 | 0.014 |
| 118 | Erbin | 0.57 | 0.000 | 118 | Adamts2 | -1.10 | 0.002 |
| 119 | Pllp | 0.57 | 0.019 | 119 | Fank1 | -1.10 | 0.025 |
| 120 | Cldn11 | 0.57 | 0.021 | 120 | Gucy2f | -1.09 | 0.040 |
| 121 | Cdc42ep1 | 0.58 | 0.013 | 121 | Ntn5 | -1.08 | 0.019 |
| 122 | Lingo3 | 0.58 | 0.023 | 122 | Ppl | -1.08 | 0.001 |
| 123 | Bcas1os1 | 0.58 | 0.029 | 123 | Chtf18 | -1.08 | 0.001 |
| 124 | Klhl13 | 0.59 | 0.002 | 124 | Cemip | -1.07 | 0.001 |
| 125 | Rgs14 | 0.59 | 0.012 | 125 | Slc16a3 | -1.06 | 0.002 |
| 126 | Aox1 | 0.59 | 0.034 | 126 | Pth1r | -1.06 | 0.008 |
| 127 | A930001C03Rik | 0.59 | 0.045 | 127 | Cbln1 | -1.06 | 0.001 |
| 128 | Gm9962 | 0.59 | 0.021 | 128 | Gm44264 | -1.05 | 0.015 |
| 129 | Sox10 | 0.60 | 0.027 | 129 | Ntsr1 | -1.05 | 0.024 |
| 130 | Pigh | 0.60 | 0.015 | 130 | Slco4a1 | -1.04 | 0.006 |
| 131 | Krcc1 | 0.60 | 0.025 | 131 | Gng13 | -1.03 | 0.005 |
| 132 | Gm1821 | 0.60 | 0.045 | 132 | Cst6 | -1.02 | 0.027 |
| 133 | Gm15966 | 0.60 | 0.005 | 133 | Necab1 | -1.02 | 0.001 |
| 134 | Acaa2 | 0.60 | 0.008 | 134 | Apaf1 | -1.02 | 0.001 |
| 135 | H2-K1 | 0.61 | 0.044 | 135 | Gm35339 | -1.01 | 0.019 |
| 136 | Ptpn3 | 0.61 | 0.022 | 136 | Sstr1 | -1.00 | 0.002 |
| 137 | Cnp | 0.61 | 0.005 | 137 | Rspo2 | -0.99 | 0.001 |
| 138 | Plekhh1 | 0.61 | 0.004 | 138 | Gm21954 | -0.98 | 0.002 |
| 139 | Plp1 | 0.61 | 0.004 | 139 | Gm1043 | -0.98 | 0.005 |
| 140 | Rbm3os | 0.62 | 0.010 | 140 | Apex2 | -0.97 | 0.017 |
| 141 | Fzd4 | 0.62 | 0.017 | 141 | Gm20421 | -0.94 | 0.008 |
| 142 | Acot2 | 0.63 | 0.025 | 142 | Lypd1 | -0.92 | 0.002 |
| 143 | Chn2 | 0.63 | 0.002 | 143 | Cntnap3 | -0.92 | 0.002 |
| 144 | Unc5b | 0.63 | 0.002 | 144 | Hpcal1 | -0.92 | 0.001 |
| 145 | Ddx49 | 0.63 | 0.020 | 145 | Map2k6 | -0.90 | 0.013 |
| 146 | Adamtsl4 | 0.63 | 0.039 | 146 | Gm26788 | -0.90 | 0.006 |
| 147 | Fa2h | 0.63 | 0.028 | 147 | Ntng2 | -0.89 | 8.5E-06 |
| 148 | Ttc30b | 0.63 | 0.026 | 148 | Zfp853 | -0.89 | 0.044 |
| 149 | Galnt6 | 0.63 | 0.013 | 149 | Hes5 | -0.89 | 0.004 |
| 150 | Sh3bgrl | 0.63 | 4.67E-04 | 150 | 4930539E08Rik | -0.89 | 0.021 |
| 151 | Anln | 0.64 | 0.005 | 151 | Mfng | -0.89 | 0.049 |
| 152 | Tmem63a | 0.64 | 0.001 | 152 | Lgi2 | -0.88 | 0.003 |
| 153 | H1f2 | 0.65 | 0.018 | 153 | Cdh23 | -0.88 | 0.034 |

| | | | | | | | |
|---|---|---|---|---|---|---|---|
| 154 | Atp11c | 0.65 | 0.044 | 154 | Hrk | -0.88 | 7.1E-05 |
| 155 | Gpr37 | 0.65 | 0.001 | 155 | Kcnc4 | -0.87 | 0.005 |
| 156 | Abhd2 | 0.65 | 0.003 | 156 | Gm13883 | -0.87 | 0.042 |
| 157 | Gm45767 | 0.65 | 0.021 | 157 | Camk2d | -0.87 | 6.2E-05 |
| 158 | Man1a | 0.66 | 0.021 | 158 | Cntnap5b | -0.87 | 0.012 |
| 159 | Trf | 0.66 | 0.003 | 159 | Cntnap5a | -0.86 | 0.004 |
| 160 | Chrm2 | 0.66 | 0.048 | 160 | Card10 | -0.86 | 0.002 |
| 161 | Mog | 0.66 | 0.005 | 161 | Rdh13 | -0.86 | 1.7E-05 |
| 162 | Zfhx4 | 0.66 | 0.005 | 162 | Kif26b | -0.86 | 0.048 |
| 163 | Wipf3 | 0.67 | 0.023 | 163 | 1700086L19Rik | -0.85 | 0.026 |
| 164 | Stim2 | 0.67 | 0.006 | 164 | Gm19744 | -0.85 | 0.025 |
| 165 | Marveld1 | 0.67 | 0.018 | 165 | Gpx3 | -0.84 | 0.011 |
| 166 | Fzd6 | 0.67 | 0.032 | 166 | Gm47205 | -0.84 | 0.019 |
| 167 | Syndig1 | 0.67 | 0.013 | 167 | C230034O21Rik | -0.82 | 0.048 |
| 168 | Zfp804a | 0.68 | 0.011 | 168 | Kcng1 | -0.82 | 0.012 |
| 169 | Phactr2 | 0.68 | 0.040 | 169 | Angpt1 | -0.81 | 0.021 |
| 170 | Mag | 0.68 | 0.007 | 170 | Il4ra | -0.81 | 0.024 |
| 171 | Cdh19 | 0.69 | 0.009 | 171 | A730098A19Rik | -0.80 | 0.042 |
| 172 | Neurod6 | 0.69 | 0.019 | 172 | Plcxd2 | -0.80 | 0.013 |
| 173 | Herc6 | 0.69 | 0.043 | 173 | Wnt7b | -0.79 | 0.001 |
| 174 | Snx33 | 0.69 | 0.012 | 174 | Cmbl | -0.79 | 0.027 |
| 175 | Cd82 | 0.69 | 0.020 | 175 | 3110056K07Rik | -0.78 | 0.036 |
| 176 | Gjc2 | 0.70 | 0.010 | 176 | Nnat | -0.78 | 0.001 |
| 177 | Aopep | 0.70 | 0.013 | 177 | Hif3a | -0.78 | 0.020 |
| 178 | S1pr5 | 0.70 | 0.007 | 178 | C2cd4c | -0.77 | 0.040 |
| 179 | Prkcq | 0.70 | 0.021 | 179 | Adcy1 | -0.76 | 0.003 |
| 180 | Gm42756 | 0.71 | 0.043 | 180 | Fosl2 | -0.76 | 0.016 |
| 181 | Efemp2 | 0.72 | 0.004 | 181 | Rom1 | -0.76 | 0.035 |
| 182 | Thsd4 | 0.72 | 0.010 | 182 | Cbfa2t3 | -0.76 | 0.006 |
| 183 | Tor3a | 0.72 | 0.019 | 183 | Gucy2e | -0.76 | 0.024 |
| 184 | Lrp10 | 0.72 | 0.005 | 184 | Ndufb9 | -0.75 | 0.002 |
| 185 | Mns1 | 0.72 | 0.026 | 185 | Rcn1 | -0.75 | 0.003 |
| 186 | Ggh | 0.74 | 0.022 | 186 | Banp | -0.75 | 0.001 |
| 187 | Hectd2os | 0.74 | 0.049 | 187 | Gm14169 | -0.75 | 0.042 |
| 188 | Prrg1 | 0.74 | 0.009 | 188 | 1500009L16Rik | -0.75 | 0.011 |
| 189 | Sox1 | 0.75 | 0.016 | 189 | Mme | -0.75 | 0.039 |
| 190 | Adra1d | 0.75 | 0.011 | 190 | Ryr1 | -0.74 | 0.010 |
| 191 | Kcnq5 | 0.76 | 0.007 | 191 | Gramd2 | -0.74 | 0.014 |
| 192 | Antxr1 | 0.76 | 0.008 | 192 | Gm45812 | -0.74 | 0.030 |
| 193 | Bcas1os2 | 0.76 | 0.004 | 193 | Kif26a | -0.74 | 0.039 |
| 194 | Fhad1 | 0.76 | 0.029 | 194 | C230057M02Rik | -0.73 | 0.008 |
| 195 | Tmem88b | 0.76 | 0.001 | 195 | Entpd2 | -0.73 | 0.036 |
| 196 | Patj | 0.77 | 0.013 | 196 | Gpr39 | -0.73 | 0.010 |
| 197 | Cdkn1c | 0.78 | 0.015 | 197 | 6530402F18Rik | -0.73 | 0.018 |
| 198 | Cd38 | 0.79 | 0.045 | 198 | Zbtb16 | -0.73 | 0.014 |
| 199 | Palmd | 0.79 | 0.037 | 199 | Lgr6 | -0.72 | 0.043 |
| 200 | Gm15651 | 0.79 | 0.042 | 200 | Cdc7 | -0.72 | 0.012 |
| 201 | Anxa4 | 0.79 | 0.014 | 201 | 1700030J22Rik | -0.72 | 0.033 |
| 202 | Gm44732 | 0.79 | 0.021 | 202 | Gm11837 | -0.72 | 0.022 |
| 203 | Met | 0.79 | 0.041 | 203 | Ddah2 | -0.71 | 0.014 |
| 204 | Gm19500 | 0.80 | 0.029 | 204 | Auts2 | -0.71 | 0.005 |
| 205 | Plcb4 | 0.80 | 0.001 | 205 | Kitl | -0.71 | 0.014 |
| 206 | Gstm7 | 0.81 | 0.016 | 206 | 4930519K11Rik | -0.70 | 0.009 |

| | | | | | | | |
|---|---|---|---|---|---|---|---|
| 207 | 1700047M11Rik | 0.81 | 0.015 | 207 | Wnt4 | -0.70 | 0.034 |
| 208 | Adamts1 | 0.81 | 0.012 | 208 | Cdkl4 | -0.70 | 0.006 |
| 209 | Arsg | 0.81 | 0.001 | 209 | Gm11973 | -0.70 | 0.010 |
| 210 | Slc12a2 | 0.81 | 1.96E-04 | 210 | Nts | -0.70 | 0.037 |
| 211 | Irgm1 | 0.82 | 0.035 | 211 | Rtbdn | -0.70 | 0.043 |
| 212 | Fbn1 | 0.82 | 0.002 | 212 | Adra2c | -0.69 | 0.005 |
| 213 | Syt6 | 0.82 | 0.006 | 213 | Wdr55 | -0.69 | 0.041 |
| 214 | Htr2c | 0.82 | 0.013 | 214 | Col23a1 | -0.69 | 0.015 |
| 215 | Xaf1 | 0.82 | 0.038 | 215 | Mmp14 | -0.69 | 0.019 |
| 216 | Nbl1 | 0.82 | 0.008 | 216 | Coro2a | -0.69 | 0.007 |
| 217 | Sfrp1 | 0.82 | 0.030 | 217 | A330076H08Rik | -0.69 | 0.047 |
| 218 | CT010467.1 | 0.82 | 3.49E-05 | 218 | Rgs16 | -0.69 | 0.016 |
| 219 | Gm16156 | 0.82 | 0.025 | 219 | Wnt5a | -0.68 | 0.018 |
| 220 | Cab39l | 0.83 | 0.002 | 220 | Asl | -0.68 | 0.008 |
| 221 | Ephx1 | 0.83 | 0.023 | 221 | Fosb | -0.68 | 0.017 |
| 222 | Emb | 0.83 | 0.008 | 222 | Rap1gap2 | -0.68 | 0.009 |
| 223 | Cntnap5c | 0.83 | 0.014 | 223 | Gmip | -0.67 | 0.045 |
| 224 | Pltp | 0.84 | 0.004 | 224 | Fzd8 | -0.67 | 0.049 |
| 225 | Fgf10 | 0.84 | 0.003 | 225 | Pak6 | -0.67 | 0.007 |
| 226 | Aspa | 0.84 | 0.002 | 226 | Cobl | -0.67 | 0.049 |
| 227 | Abcb1b | 0.85 | 0.024 | 227 | Lhx2 | -0.66 | 0.001 |
| 228 | Pde3a | 0.85 | 0.042 | 228 | Grem2 | -0.66 | 0.019 |
| 229 | Enpp6 | 0.85 | 0.003 | 229 | Krt77 | -0.66 | 0.026 |
| 230 | Lats2 | 0.86 | 0.007 | 230 | Anks6 | -0.65 | 0.008 |
| 231 | Nqo1 | 0.86 | 0.038 | 231 | Ntng1 | -0.65 | 0.007 |
| 232 | Prdm16 | 0.86 | 0.017 | 232 | Tmsb10 | -0.65 | 0.016 |
| 233 | Rab11fip1 | 0.86 | 0.038 | 233 | Il17d | -0.65 | 0.044 |
| 234 | Rnf152 | 0.87 | 0.019 | 234 | Casz1 | -0.65 | 0.022 |
| 235 | Rnf182 | 0.87 | 0.012 | 235 | Klhdc8a | -0.65 | 0.022 |
| 236 | Cdr2 | 0.87 | 0.023 | 236 | Cdr1 | -0.64 | 0.018 |
| 237 | Sowahc | 0.87 | 0.001 | 237 | Cav2 | -0.64 | 0.003 |
| 238 | Car14 | 0.88 | 0.005 | 238 | Rims4 | -0.64 | 0.043 |
| 239 | 2210411M09Rik | 0.89 | 0.004 | 239 | Gm44354 | -0.63 | 0.036 |
| 240 | Tcn2 | 0.90 | 0.008 | 240 | Kiss1r | -0.63 | 0.038 |
| 241 | Tmem125 | 0.90 | 0.005 | 241 | Shisa9 | -0.63 | 0.017 |
| 242 | Vat1l | 0.90 | 0.002 | 242 | Smc2 | -0.62 | 0.039 |
| 243 | Gm42418 | 0.90 | 7.42E-06 | 243 | AB041806 | -0.62 | 0.046 |
| 244 | Prr5l | 0.91 | 0.002 | 244 | Gm10736 | -0.62 | 0.013 |
| 245 | Arhgap15 | 0.91 | 0.042 | 245 | 2900052N01Rik | -0.62 | 0.028 |
| 246 | Gm36279 | 0.92 | 0.036 | 246 | Tmem145 | -0.62 | 0.016 |
| 247 | Fzd7 | 0.94 | 0.012 | 247 | Gm50301 | -0.62 | 0.018 |
| 248 | Lratd2 | 0.94 | 0.018 | 248 | Gm13375 | -0.61 | 0.020 |
| 249 | Col25a1 | 0.94 | 0.002 | 249 | Iffo2 | -0.61 | 0.004 |
| 250 | Lars2 | 0.94 | 1.10E-04 | 250 | Kcnj16 | -0.61 | 0.015 |
| 251 | Sod3 | 0.94 | 0.005 | 251 | Nos1 | -0.61 | 0.005 |
| 252 | Hfe | 0.94 | 0.037 | 252 | Exoc3l | -0.61 | 0.041 |
| 253 | Cyp27a1 | 0.95 | 0.023 | 253 | Vstm2b | -0.61 | 0.025 |
| 254 | Dlx6 | 0.95 | 0.046 | 254 | Akain1 | -0.60 | 0.040 |
| 255 | Cachd1 | 0.95 | 0.002 | 255 | Jun | -0.60 | 0.024 |
| 256 | Prss23 | 0.96 | 0.003 | 256 | Gpr26 | -0.60 | 0.007 |
| 257 | Gask1b | 0.96 | 0.031 | 257 | Map2k3 | -0.60 | 0.048 |
| 258 | Trim59 | 0.96 | 1.83E-05 | 258 | Srrm4 | -0.60 | 0.011 |
| 259 | Bmp6 | 0.97 | 0.032 | 259 | Sdf2l1 | -0.60 | 0.048 |

| | | | | | | | |
|---|---|---|---|---|---|---|---|
| 260 | Saxo2 | 0.97 | 0.049 | 260 | Grk3 | -0.60 | 0.008 |
| 261 | Ifitm3 | 0.97 | 0.031 | 261 | Rnf207 | -0.60 | 0.037 |
| 262 | Slc9a4 | 0.98 | 0.038 | 262 | Igfbp5 | -0.59 | 0.012 |
| 263 | Ccdc122 | 0.98 | 0.048 | 263 | Pisd-ps1 | -0.59 | 0.008 |
| 264 | Ankub1 | 0.99 | 0.036 | 264 | Fn1 | -0.59 | 0.039 |
| 265 | Tafa1 | 1.00 | 0.001 | 265 | D430041D05Rik | -0.58 | 0.008 |
| 266 | Tmem98 | 1.01 | 0.003 | 266 | Atn1 | -0.58 | 0.044 |
| 267 | Acss3 | 1.02 | 0.036 | 267 | Tspan9 | -0.58 | 0.004 |
| 268 | Hspb6 | 1.02 | 0.015 | 268 | 1700001L19Rik | -0.57 | 0.049 |
| 269 | Homer3 | 1.03 | 0.006 | 269 | Hk2 | -0.57 | 0.048 |
| 270 | Ky | 1.03 | 0.035 | 270 | Rnf217 | -0.57 | 0.040 |
| 271 | Gm43109 | 1.04 | 0.043 | 271 | Gm17092 | -0.57 | 0.022 |
| 272 | Trim21 | 1.05 | 0.035 | 272 | Mras | -0.57 | 0.013 |
| 273 | Abi3bp | 1.05 | 0.039 | 273 | Limk1 | -0.57 | 0.031 |
| 274 | Gprc5c | 1.05 | 0.023 | 274 | Zmym1 | -0.57 | 0.019 |
| 275 | Rsph4a | 1.06 | 0.046 | 275 | Ulk2 | -0.56 | 0.001 |
| 276 | St6galnac2 | 1.08 | 0.027 | 276 | Smad3 | -0.56 | 0.004 |
| 277 | 4930412C18Rik | 1.09 | 0.046 | 277 | Gm23134 | -0.56 | 0.027 |
| 278 | Epyc | 1.09 | 0.034 | 278 | Slc6a7 | -0.56 | 0.009 |
| 279 | Ucp2 | 1.09 | 0.001 | 279 | Fgf20 | -0.56 | 0.042 |
| 280 | Sema3e | 1.10 | 5.17E-05 | 280 | Shc3 | -0.55 | 0.034 |
| 281 | Zfp119b | 1.10 | 0.029 | 281 | Tiam1 | -0.55 | 0.014 |
| 282 | Zfhx3 | 1.11 | 0.004 | 282 | Sox11 | -0.55 | 0.038 |
| 283 | Slc4a2 | 1.11 | 0.001 | 283 | Usp35 | -0.55 | 0.028 |
| 284 | Slc31a1 | 1.12 | 0.002 | 284 | Dpysl4 | -0.55 | 0.014 |
| 285 | Gm23935 | 1.12 | 3.87E-06 | 285 | Man1c1 | -0.55 | 0.048 |
| 286 | Gm26684 | 1.13 | 0.049 | 286 | 1110008P14Rik | -0.55 | 0.044 |
| 287 | Pih1d2 | 1.14 | 0.039 | 287 | Stx8 | -0.54 | 0.024 |
| 288 | Cgnl1 | 1.14 | 0.001 | 288 | Gm26953 | -0.54 | 0.026 |
| 289 | Serpinb8 | 1.14 | 0.022 | 289 | Miat | -0.54 | 0.042 |
| 290 | Aim2 | 1.16 | 0.034 | 290 | Ccdc136 | -0.53 | 0.006 |
| 291 | Rem2 | 1.16 | 0.027 | 291 | Slc24a4 | -0.53 | 0.033 |
| 292 | Cfap61 | 1.17 | 0.037 | 292 | Pitpnm3 | -0.53 | 0.008 |
| 293 | Drd2 | 1.18 | 0.022 | 293 | Inha | -0.53 | 0.030 |
| 294 | Steap2 | 1.18 | 0.002 | 294 | Adcyap1r1 | -0.53 | 0.009 |
| 295 | Gm34256 | 1.18 | 0.049 | 295 | Dio2 | -0.52 | 0.032 |
| 296 | A830012C17Rik | 1.18 | 0.015 | 296 | Pcsk1 | -0.52 | 0.038 |
| 297 | Ifit3b | 1.19 | 0.004 | 297 | Secisbp2 | -0.52 | 0.024 |
| 298 | St8sia6 | 1.19 | 0.031 | 298 | Myh7 | -0.51 | 0.034 |
| 299 | C130074G19Rik | 1.19 | 2.95E-04 | 299 | Fam124a | -0.51 | 0.044 |
| 300 | Gm17509 | 1.19 | 0.003 | 300 | Engase | -0.51 | 0.048 |
| 301 | Inmt | 1.20 | 0.049 | 301 | Arhgef19 | -0.51 | 0.016 |
| 302 | Gm19935 | 1.20 | 0.031 | 302 | Fxyd7 | -0.51 | 0.030 |
| 303 | Tal1 | 1.25 | 0.036 | 303 | Atp2b4 | -0.51 | 0.038 |
| 304 | Ifi27 | 1.26 | 2.18E-04 | 304 | Klf12 | -0.50 | 0.048 |
| 305 | Epn3 | 1.26 | 0.009 | 305 | Arpp21 | -0.50 | 0.020 |
| 306 | Gm5112 | 1.30 | 0.028 | 306 | Txnl4a | -0.50 | 0.031 |
| 307 | Ifi35 | 1.32 | 0.026 | 307 | Csf2ra | -0.50 | 0.028 |
| 308 | 4933406C10Rik | 1.32 | 0.042 | 308 | Plxna3 | -0.50 | 0.040 |
| 309 | Gm45472 | 1.32 | 0.011 | 309 | Cyp4f15 | -0.50 | 0.050 |
| 310 | Msx1 | 1.33 | 0.001 | 310 | Kirrel3 | -0.50 | 0.046 |
| 311 | H2-Aa | 1.33 | 0.036 | 311 | Fbxo2 | -0.49 | 0.027 |
| 312 | Rtp4 | 1.34 | 0.021 | 312 | Tafa2 | -0.49 | 0.017 |

| | | | | | | | |
|---|---|---|---|---|---|---|---|
| 313 | Gm24270 | 1.34 | 1.63E-05 | 313 | Cdk18 | -0.49 | 0.036 |
| 314 | Npr1 | 1.36 | 0.003 | 314 | Mcf2l | -0.49 | 0.005 |
| 315 | Tmem54 | 1.36 | 0.027 | 315 | Ssh3 | -0.49 | 0.030 |
| 316 | BC067074 | 1.37 | 0.007 | 316 | Rab3b | -0.49 | 0.024 |
| 317 | 4921539H07Rik | 1.37 | 0.011 | 317 | Rps28 | -0.48 | 0.048 |
| 318 | Ifi27l2a | 1.39 | 0.014 | 318 | Fgd6 | -0.48 | 0.037 |
| 319 | Clec18a | 1.39 | 0.025 | 319 | Phka2 | -0.48 | 0.036 |
| 320 | Nudt12os | 1.42 | 0.018 | 320 | Elk1 | -0.48 | 0.029 |
| 321 | Baiap2l1 | 1.42 | 0.023 | 321 | Uba6 | -0.48 | 0.019 |
| 322 | Capsl | 1.42 | 0.047 | 322 | Kcnip3 | -0.48 | 0.026 |
| 323 | Gm26644 | 1.45 | 0.015 | 323 | Sarm1 | -0.48 | 0.009 |
| 324 | 1700007F19Rik | 1.45 | 0.040 | 324 | Prkab2 | -0.47 | 0.025 |
| 325 | Krt8 | 1.47 | 0.020 | 325 | Rps29 | -0.47 | 0.032 |
| 326 | Slc2a12 | 1.47 | 1.22E-04 | 326 | Ypel4 | -0.47 | 0.010 |
| 327 | Ccdc113 | 1.47 | 0.025 | 327 | Sh3bgrl2 | -0.47 | 0.015 |
| 328 | Rsph1 | 1.47 | 0.017 | 328 | Tmem150c | -0.47 | 0.026 |
| 329 | Creb5 | 1.47 | 0.000 | 329 | Fam241b | -0.46 | 0.043 |
| 330 | Ccdc187 | 1.48 | 0.008 | 330 | 1700037H04Rik | -0.46 | 0.009 |
| 331 | A930017K11Rik | 1.50 | 0.012 | 331 | Gm14817 | -0.46 | 0.034 |
| 332 | Elovl7 | 1.50 | 6.4E-05 | 332 | Paqr7 | -0.46 | 0.004 |
| 333 | Tmem184a | 1.50 | 0.036 | 333 | Syt17 | -0.46 | 0.028 |
| 334 | Sspnos | 1.50 | 0.027 | 334 | Rpl19 | -0.45 | 0.022 |
| 335 | Sgms2 | 1.51 | 0.005 | 335 | Psd3 | -0.45 | 0.027 |
| 336 | Gm26917 | 1.53 | 0.046 | 336 | Nhsl2 | -0.45 | 0.047 |
| 337 | Slc37a2 | 1.54 | 0.002 | 337 | Tubb3 | -0.44 | 0.044 |
| 338 | Ifit3 | 1.54 | 7.18E-05 | 338 | Rhobtb2 | -0.44 | 0.050 |
| 339 | Frem1 | 1.55 | 0.003 | 339 | Kif1a | -0.44 | 0.025 |
| 340 | Postn | 1.55 | 0.043 | 340 | Trim11 | -0.44 | 0.036 |
| 341 | Gbp3 | 1.56 | 4.99E-04 | 341 | Itga3 | -0.44 | 0.034 |
| 342 | Ace | 1.56 | 0.037 | 342 | Sgsm2 | -0.44 | 0.007 |
| 343 | Fbln7 | 1.58 | 0.047 | 343 | Carmil3 | -0.44 | 0.033 |
| 344 | Ptgs2 | 1.58 | 0.001 | 344 | Gpsm1 | -0.43 | 0.011 |
| 345 | Trim30a | 1.58 | 0.003 | 345 | Ramp1 | -0.43 | 0.031 |
| 346 | 4932438H23Rik | 1.61 | 0.030 | 346 | Cdh13 | -0.43 | 0.048 |
| 347 | Gm11651 | 1.63 | 0.001 | 347 | Gria4 | -0.43 | 0.048 |
| 348 | Strip2 | 1.64 | 0.009 | 348 | Gm9866 | -0.43 | 0.050 |
| 349 | Dnaic2 | 1.64 | 0.009 | 349 | Cacna1h | -0.43 | 0.036 |
| 350 | Adgrd1 | 1.64 | 0.003 | 350 | Tbc1d8 | -0.43 | 0.045 |
| 351 | Angptl2 | 1.66 | 0.001 | 351 | Dzank1 | -0.43 | 0.049 |
| 352 | Dnah11 | 1.67 | 0.037 | 352 | D430042O09Rik | -0.42 | 0.035 |
| 353 | Lrrc23 | 1.67 | 0.023 | 353 | D430019H16Rik | -0.42 | 0.041 |
| 354 | Rbm47 | 1.68 | 0.004 | 354 | Dhx29 | -0.42 | 0.047 |
| 355 | Ifit1 | 1.68 | 0.001 | 355 | Kifc2 | -0.42 | 0.041 |
| 356 | Tekt1 | 1.68 | 0.018 | 356 | Mapk3 | -0.42 | 0.014 |
| 357 | Ubxn10 | 1.69 | 0.016 | 357 | Rilpl1 | -0.42 | 0.027 |
| 358 | Fap | 1.69 | 0.002 | 358 | Crocc | -0.41 | 0.016 |
| 359 | Twist1 | 1.70 | 0.048 | 359 | Unc13b | -0.41 | 0.021 |
| 360 | Tjp3 | 1.71 | 0.040 | 360 | Prag1 | -0.40 | 0.037 |
| 361 | 1500015L24Rik | 1.72 | 0.003 | 361 | Kif5a | -0.40 | 0.049 |
| 362 | Oasl2 | 1.73 | 0.001 | 362 | Ablim2 | -0.40 | 0.024 |
| 363 | Sap30bpos | 1.76 | 0.041 | 363 | Gfod1 | -0.40 | 0.028 |
| 364 | Bdh2 | 1.77 | 0.012 | 364 | Gap43 | -0.40 | 0.032 |
| 365 | Pcolce2 | 1.78 | 0.001 | 365 | Mta3 | -0.40 | 0.042 |

| | | | | | | | |
|---|---|---|---|---|---|---|---|
| 366 | Sulf1 | 1.79 | 0.027 | 366 | Col4a2 | -0.39 | 0.046 |
| 367 | C1ra | 1.79 | 0.007 | 367 | Cadm1 | -0.39 | 0.024 |
| 368 | Tc2n | 1.81 | 0.027 | 368 | Pon2 | -0.39 | 0.049 |
| 369 | Stk33 | 1.81 | 0.010 | 369 | Dgcr6 | -0.39 | 0.046 |
| 370 | Gm20444 | 1.83 | 8.6E-05 | 370 | Dlg5 | -0.39 | 0.024 |
| 371 | Calml4 | 1.85 | 0.008 | 371 | Pxdn | -0.39 | 0.020 |
| 372 | Pla2g5 | 1.85 | 0.002 | 372 | Mxd4 | -0.39 | 0.014 |
| 373 | Sema3b | 1.86 | 0.029 | 373 | Abcc8 | -0.39 | 0.049 |
| 374 | Ccdc162 | 1.86 | 0.013 | 374 | Rimbp2 | -0.38 | 0.037 |
| 375 | Large2 | 1.87 | 0.020 | 375 | Col4a1 | -0.38 | 0.044 |
| 376 | Odf3b | 1.87 | 0.021 | 376 | Nyap1 | -0.38 | 0.038 |
| 377 | Slitrk6 | 1.88 | 0.024 | 377 | Hmbox1 | -0.38 | 0.026 |
| 378 | Irf7 | 1.88 | 3.0E-05 | 378 | Abhd8 | -0.38 | 0.040 |
| 379 | Gm46658 | 1.89 | 0.011 | 379 | Ulk1 | -0.38 | 0.042 |
| 380 | Pifo | 1.90 | 0.026 | 380 | Evl | -0.37 | 0.038 |
| 381 | Wfikkn2 | 1.91 | 0.001 | 381 | Dtnb | -0.37 | 0.030 |
| 382 | 4933416M07Rik | 1.92 | 0.048 | 382 | Tef | -0.37 | 0.037 |
| 383 | Rgs22 | 1.92 | 0.034 | 383 | Tspoap1 | -0.37 | 0.045 |
| 384 | Gm44250 | 1.93 | 0.001 | 384 | Tbc1d9b | -0.36 | 0.046 |
| 385 | Pnlip | 1.94 | 0.038 | 385 | Zfr2 | -0.36 | 0.047 |
| 386 | Maats1 | 1.94 | 0.020 | 386 | Elavl3 | -0.35 | 0.036 |
| 387 | Lgals3bp | 1.94 | 0.007 | 387 | Sh2d5 | -0.35 | 0.028 |
| 388 | Col4a4 | 1.95 | 0.007 | 388 | Ets2 | -0.35 | 0.044 |
| 389 | Krt18 | 1.95 | 0.001 | 389 | Urgcp | -0.34 | 0.049 |
| 390 | Slfn9 | 1.95 | 0.024 | 390 | Crtac1 | -0.32 | 0.047 |
| 391 | Armc3 | 1.96 | 0.018 | 391 | R3hdm4 | -0.32 | 0.032 |
| 392 | Gm4951 | 1.96 | 0.025 | 392 | Abcc5 | -0.32 | 0.048 |
| 393 | A530053G22Rik | 1.97 | 0.022 | 393 | Rian | -0.30 | 0.048 |
| 394 | Gm43046 | 1.97 | 0.032 | 394 | Timp2 | -0.30 | 0.032 |
| 395 | Eps8l1 | 1.98 | 7.0E-08 | | | | |
| 396 | Irx3 | 1.98 | 0.034 | | | | |
| 397 | Ccdc146 | 2.02 | 0.030 | | | | |
| 398 | Usp18 | 2.03 | 0.002 | | | | |
| 399 | 2010001K21Rik | 2.03 | 0.041 | | | | |
| 400 | Ifi44 | 2.04 | 0.010 | | | | |
| 401 | Cldn3 | 2.04 | 0.040 | | | | |
| 402 | Col6a5 | 2.05 | 0.025 | | | | |
| 403 | Otx2 | 2.07 | 0.010 | | | | |
| 404 | Gm19963 | 2.08 | 0.036 | | | | |
| 405 | Spag16 | 2.10 | 0.003 | | | | |
| 406 | Gm16090 | 2.10 | 0.025 | | | | |
| 407 | Armc4 | 2.11 | 0.042 | | | | |
| 408 | Magel2 | 2.11 | 0.003 | | | | |
| 409 | Pon3 | 2.12 | 4.1E-04 | | | | |
| 410 | Serpinb1b | 2.12 | 0.015 | | | | |
| 411 | Npffr1 | 2.15 | 0.003 | | | | |
| 412 | C230086J09Rik | 2.15 | 0.012 | | | | |
| 413 | Nek5 | 2.17 | 0.036 | | | | |
| 414 | Dnah6 | 2.17 | 0.014 | | | | |
| 415 | Fam81b | 2.18 | 0.039 | | | | |
| 416 | Bok | 2.21 | 0.016 | | | | |
| 417 | Wfdc2 | 2.21 | 0.018 | | | | |
| 418 | Col8a2 | 2.21 | 0.028 | | | | |

| | | | | | | | |
|---|---|---|---|---|---|---|---|
| 419 | Bst2 | 2.21 | 2.9E-05 | | | | |
| 420 | Sp8 | 2.22 | 0.002 | | | | |
| 421 | Col9a3 | 2.22 | 0.005 | | | | |
| 422 | Pld5 | 2.22 | 0.001 | | | | |
| 423 | Scara5 | 2.23 | 0.021 | | | | |
| 424 | Ttc21a | 2.24 | 0.002 | | | | |
| 425 | Ttll6 | 2.24 | 0.022 | | | | |
| 426 | Zic1 | 2.26 | 0.045 | | | | |
| 427 | Crocc2 | 2.26 | 0.029 | | | | |
| 428 | Clic6 | 2.28 | 0.001 | | | | |
| 429 | Slc16a8 | 2.28 | 0.002 | | | | |
| 430 | Prlr | 2.30 | 0.010 | | | | |
| 431 | E230013L22Rik | 2.31 | 0.043 | | | | |
| 432 | Pou4f1 | 2.34 | 0.034 | | | | |
| 433 | Drc7 | 2.34 | 2.5E-05 | | | | |
| 434 | Rab20 | 2.36 | 0.002 | | | | |
| 435 | Cdh3 | 2.37 | 0.034 | | | | |
| 436 | Zfp979 | 2.40 | 0.018 | | | | |
| 437 | Apol9a | 2.41 | 0.014 | | | | |
| 438 | Ebf2 | 2.42 | 0.016 | | | | |
| 439 | 2900040C04Rik | 2.42 | 0.001 | | | | |
| 440 | Slc39a4 | 2.43 | 0.005 | | | | |
| 441 | Rdh5 | 2.43 | 0.001 | | | | |
| 442 | Zic3 | 2.46 | 2.2E-04 | | | | |
| 443 | Myb | 2.46 | 0.008 | | | | |
| 444 | Kl | 2.46 | 0.001 | | | | |
| 445 | Ebf3 | 2.48 | 6.34E-05 | | | | |
| 446 | Tctex1d4 | 2.48 | 0.002 | | | | |
| 447 | Pate2 | 2.49 | 0.039 | | | | |
| 448 | Gm43398 | 2.49 | 0.001 | | | | |
| 449 | Cdh26 | 2.49 | 0.044 | | | | |
| 450 | Prr32 | 2.51 | 4.8E-04 | | | | |
| 451 | Ptprh | 2.53 | 0.002 | | | | |
| 452 | Tgtp1 | 2.56 | 0.001 | | | | |
| 453 | Sult1c1 | 2.60 | 0.020 | | | | |
| 454 | Adgb | 2.60 | 0.031 | | | | |
| 455 | Cfap206 | 2.64 | 0.006 | | | | |
| 456 | Capn11 | 2.64 | 0.010 | | | | |
| 457 | AC151602.1 | 2.66 | 0.011 | | | | |
| 458 | Grhl2 | 2.67 | 0.014 | | | | |
| 459 | Wdr63 | 2.67 | 0.007 | | | | |
| 460 | 4833427G06Rik | 2.69 | 0.019 | | | | |
| 461 | Trpv4 | 2.70 | 2.2E-06 | | | | |
| 462 | Ube4bos2 | 2.71 | 0.040 | | | | |
| 463 | Lbp | 2.73 | 0.003 | | | | |
| 464 | Otx2os1 | 2.74 | 0.014 | | | | |
| 465 | Enpp2 | 2.76 | 1.4E-04 | | | | |
| 466 | Wdr86 | 2.76 | 5.82E-05 | | | | |
| 467 | Tmem154 | 2.76 | 0.037 | | | | |
| 468 | Ccdc170 | 2.77 | 0.016 | | | | |
| 469 | Sfrp5 | 2.78 | 1.0E-04 | | | | |
| 470 | Smim22 | 2.78 | 0.028 | | | | |
| 471 | Haglr | 2.83 | 0.037 | | | | |

| | | | | | | | |
|---|---|---|---|---|---|---|---|
| 472 | 3300002A11Rik | 2.84 | 0.012 | | | | |
| 473 | Slc14a2 | 2.85 | 0.023 | | | | |
| 474 | Nat8f7 | 2.90 | 0.026 | | | | |
| 475 | Gm49677 | 2.90 | 0.036 | | | | |
| 476 | Slc28a3 | 2.91 | 0.018 | | | | |
| 477 | Serpina3n | 2.91 | 1.6E-33 | | | | |
| 478 | Aqp1 | 2.93 | 0.002 | | | | |
| 479 | Pcdhb1 | 2.94 | 0.028 | | | | |
| 480 | Gm15958 | 3.06 | 0.025 | | | | |
| 481 | Cltrn | 3.09 | 0.023 | | | | |
| 482 | Gm15873 | 3.09 | 0.008 | | | | |
| 483 | Wdr72 | 3.11 | 0.003 | | | | |
| 484 | Gm47175 | 3.11 | 0.044 | | | | |
| 485 | Iyd | 3.12 | 0.008 | | | | |
| 486 | Hjv | 3.12 | 0.049 | | | | |
| 487 | Gm43081 | 3.12 | 0.044 | | | | |
| 488 | Gm15810 | 3.17 | 0.013 | | | | |
| 489 | Slco1a5 | 3.18 | 0.024 | | | | |
| 490 | Sult1c2 | 3.22 | 0.001 | | | | |
| 491 | Car9 | 3.25 | 0.015 | | | | |
| 492 | Cd109 | 3.25 | 0.021 | | | | |
| 493 | Glra1 | 3.25 | 0.042 | | | | |
| 494 | Sostdc1 | 3.26 | 1.1E-09 | | | | |
| 495 | Lmx1a | 3.27 | 0.002 | | | | |
| 496 | Daw1 | 3.27 | 0.043 | | | | |
| 497 | Col8a1 | 3.32 | 3.48E-04 | | | | |
| 498 | 4933429O19Rik | 3.34 | 0.005 | | | | |
| 499 | Cldn9 | 3.37 | 0.044 | | | | |
| 500 | Slc4a5 | 3.40 | 4.9E-04 | | | | |
| 501 | Cfap77 | 3.42 | 0.025 | | | | |
| 502 | F5 | 3.43 | 0.001 | | | | |
| 503 | Uncx | 3.43 | 0.048 | | | | |
| 504 | Dmbx1 | 3.44 | 0.007 | | | | |
| 505 | Kcne2 | 3.50 | 4.4E-07 | | | | |
| 506 | Slc6a19 | 3.53 | 0.012 | | | | |
| 507 | Oca2 | 3.54 | 1.19E-04 | | | | |
| 508 | Mfrp | 3.60 | 3.2E-05 | | | | |
| 509 | Gm44027 | 3.61 | 0.043 | | | | |
| 510 | Barhl1 | 3.61 | 0.012 | | | | |
| 511 | Caps2 | 3.62 | 0.012 | | | | |
| 512 | Defb11 | 3.63 | 0.012 | | | | |
| 513 | BC051019 | 3.64 | 0.022 | | | | |
| 514 | Kcnj13 | 3.68 | 9.9E-05 | | | | |
| 515 | Lhfpl1 | 3.69 | 0.043 | | | | |
| 516 | Gm16548 | 3.70 | 0.001 | | | | |
| 517 | Oscar | 3.72 | 1.4E-04 | | | | |
| 518 | Shox2 | 3.73 | 0.045 | | | | |
| 519 | Cldn2 | 3.73 | 3.0E-04 | | | | |
| 520 | Nutm1 | 3.78 | 0.038 | | | | |
| 521 | 1700012B09Rik | 3.80 | 0.017 | | | | |
| 522 | Arhgap33os | 3.81 | 0.029 | | | | |
| 523 | Sox14 | 3.82 | 0.020 | | | | |
| 524 | Gm37965 | 3.83 | 0.045 | | | | |

| | | | | | | | |
|---|---|---|---|---|---|---|---|
| 525 | Gata3 | 3.83 | 0.010 | | | | |
| 526 | Styk1 | 3.83 | 0.009 | | | | |
| 527 | Glp1r | 3.84 | 0.008 | | | | |
| 528 | Steap1 | 3.86 | 8.7E-07 | | | | |
| 529 | Gm37738 | 3.86 | 0.029 | | | | |
| 530 | Ecrg4 | 3.89 | 3.0E-04 | | | | |
| 531 | Cldn22 | 3.89 | 0.002 | | | | |
| 532 | 4921534H16Rik | 3.91 | 0.046 | | | | |
| 533 | Igfals | 3.92 | 0.020 | | | | |
| 534 | Lhx1os | 3.97 | 0.036 | | | | |
| 535 | Ubash3a | 4.00 | 0.025 | | | | |
| 536 | 1700024G13Rik | 4.01 | 0.004 | | | | |
| 537 | Folr1 | 4.05 | 1.6E-06 | | | | |
| 538 | Chrnb4 | 4.05 | 0.004 | | | | |
| 539 | Gm21680 | 4.10 | 0.047 | | | | |
| 540 | Gm44625 | 4.12 | 0.046 | | | | |
| 541 | Gm17743 | 4.14 | 0.042 | | | | |
| 542 | Scn10a | 4.14 | 0.028 | | | | |
| 543 | Gm32468 | 4.15 | 0.029 | | | | |
| 544 | Dmrtb1 | 4.16 | 0.030 | | | | |
| 545 | Pebp4 | 4.20 | 0.044 | | | | |
| 546 | 1700030C10Rik | 4.27 | 0.008 | | | | |
| 547 | Adra2b | 4.31 | 0.026 | | | | |
| 548 | 4921523L03Rik | 4.31 | 0.007 | | | | |
| 549 | Pou4f2 | 4.33 | 0.014 | | | | |
| 550 | Gm45131 | 4.33 | 0.032 | | | | |
| 551 | Ttr | 4.39 | 1.0E-04 | | | | |
| 552 | Sln | 4.45 | 0.001 | | | | |
| 553 | Gm47461 | 4.45 | 0.022 | | | | |
| 554 | Gm10860 | 4.49 | 0.042 | | | | |
| 555 | Gm34035 | 4.51 | 0.030 | | | | |
| 556 | Olfr1507 | 4.70 | 0.012 | | | | |
| 557 | Gm2379 | 4.74 | 0.033 | | | | |
| 558 | Pon1 | 4.80 | 0.002 | | | | |
| 559 | Tmem72 | 4.84 | 0.001 | | | | |
| 560 | Slc26a3 | 4.87 | 9.1E-03 | | | | |
| 561 | Gm42766 | 4.91 | 0.009 | | | | |
| 562 | 7120432I05Rik | 4.91 | 0.008 | | | | |
| 563 | Omp | 5.33 | 1.3E-04 | | | | |
| 564 | Tmprss11a | 5.78 | 7.1E-06 | | | | |
| 565 | Gm5815 | 6.05 | 1.0E-04 | | | | |
