# Supplementary material for: Age- and ApoE Genotype-Dependent Transcriptomic Responses to O3 in the Hippocampus of Mice
Source: Int J Mol Sci. 2025 Mar 7;26(6):2407. doi: 10.3390/ijms26062407 (PMC11942628; doi:10.3390/ijms26062407)
Supplement: Supplementary file 1 [file ijms-26-02407-s001.zip › Supplementary Table S4a E4_vs_E3 KEGG pathway.pdf]

**Supplementary Table S4a E4_vs_E3 KEGG pathway**

| **Upregulated KEGG pathway** | **Upregulated genes** | |
|---|---|---|
| Taurine and hypotaurine metabolism | Gad2, Acnat1, Ggt5, Ggt6 | 4 |
| Fatty acid elongation | Acaa2, Acot1, Elovl7, Acot2, Acot5 | 5 |
| TNF signaling pathway | Cflar, Nod2, Cyld, Traf1, Vegfc, Csf1, Ptgs2, Creb3l2, Creb3l1, Creb5, Vcam1, Cxcl5, Irf1, Frmd8 | 14 |
| Leukocyte transendothelial migration | Ocln, Ezr, Myl12a, Ptk2b, Cldn14, Cldn9, F11r, Cldn3, Rap1a, Cldn19, Vcam1, Mmp2, Cldn11, Ctnna1, Cldn2, Vav3, Cldn1 | 17 |
| Neuroactive ligand-receptor interaction | Pdyn, Gria1, Htr2c, Drd3, S1pr5, Prlr, Avpr1b, Lhb, Grin2b, Npb, Adra2b, Glp1r, Tacr1, Lpar3, Tbxa2r, Tac4, Cort, Npffr1, Gabrp, Chrnb4, Bdkrb1, Chrna3, Gh, Drd2, Gpr50, Mc3r, C3, Sstr5, Vipr2, Oprk1, Adora2a, Npy2r, Lpar1, Crhr2, Gabrr2, Cckar, Avpr1a, Penk, Lpar6, Tspo, Prl, Adra1d, Gabra5, Htr1d, Lepr, Ptafr, Oxt | 47 |
| **Downregulated KEGG pathway** | **Downregulated genes** | |
| Inflammatory mediator regulation of TRP channels | Map2k6, Itpr3, Pla2g4b, Pik3cd, Mapk13, Cyp2j8, Alox12e, Gnas, Hrh1, Il1b, Adcy4, Adcy7, Mapk11, Camk2d | 14 |
| Neuroactive ligand-receptor interaction | Hrh2, S1pr4, Htr6, Tacr3, Vipr1, Nmbr, Grm4, Grm2, Htr5a, Hrh1, Galr2, Edn1, Chrna4, Rxfp1, Calca, Calcb, Sstr1, Ghrl, Kiss1r, Pth1r, Tacr2, Crhr1, Vip | 23 |
| Lipid and atherosclerosis | Bid, Pparg, Map2k6, Pik3cd, Mapk13, Cyp2j8, Apaf1, Il1b, Nox1, Arhgef1, Casp1, Mapk11, Camk2d | 13 |
| TNF signaling pathway | Map2k6, Rhbdf1, Pik3cd, Mapk13, Edn1, Il1b, Ripk3, Mapk11 | 8 |
| Neurotrophin signaling pathway | Arhgdig, Sh2b2, Pik3cd, Mapk13, Shc2, Matk, Mapk11, Camk2d | 8 |
