# Supplementary material for: Age- and ApoE Genotype-Dependent Transcriptomic Responses to O3 in the Hippocampus of Mice
Source: Int J Mol Sci. 2025 Mar 7;26(6):2407. doi: 10.3390/ijms26062407 (PMC11942628; doi:10.3390/ijms26062407)
Supplement: Supplementary file 1 [file ijms-26-02407-s001.zip › Supplementary Table S4b 17M_vs_3M KEGG pathway.pdf]

Supplementary Table S4b 17M_vs_3M KEGG pathway

**Upregulated KEGG pathway**	**Upregulated genes**	
Relaxin signaling pathway	Slc8a1, Atp2b3, Met, Nos2, Grin3a, Cacna1g, Ppp3r2, Drd5	8
Retrograde endocannabinoid signaling	Htr1b, Nmbr, Chrna5, Htr1a, Mc5r, Grin3a, Npbwr1, Nts, Sstr4, Drd5	9
Sphingolipid signaling pathway	S1pr5, Sphk1, Rac2, Acer2, Mapk12, Tnf, Bdkrb2, Sptssb	8
Chemokine signaling pathway	Dock2, Rac2, Ncf1, Ccl3, Hck, Gnb3, Ccl12, Pik3r5, Gng11, Gngt2, Cxcl12	11
PPAR signaling pathway	Cyp27a1, Plin1, Fabp4, Ehhadh, Adipoq, Olr1, Plin4	7
Lipid and atherosclerosis	Irf7, Nlrp3, Ncf1, Mapk12, Tnfsf10, Cyba, Ccl3, Tnf, Ccl12, Pycard, Olr1, Cd14, Lbp	13
Toll-like receptor signaling pathway	Irf7, Mapk12, Ccl3, Tnf, Cd86, Spp1, Cd14, Lbp	8
AGE-RAGE signaling pathway in diabetic complications	Col4a3, Col4a4, Serpine1, Mapk12, Tnf, Il1a, Agt, Ccl12	8
Inflammatory bowel disease	H2-Oa, Tnf, Il21r, Il1a, Rorc, Il2rg	6
Leukocyte transendothelial migration	Itgb2, Cldn22, Rac2, Ncf1, Mapk12, Cyba, Cldn2, Myl2, Cxcl12	9
Neuroactive ligand-receptor interaction	S1pr5, Avpr1b, Lhb, Gabrr1, Drd4, Mc1r, C3ar1, Adm, Gabrd, Gh, F2rl1, Trh, Pomc, Bdkrb2, Agt, Rln3, Gabrr2, Prl, Chrna6, Gipr, Ptgir	21
RIG-I-like receptor signaling pathway	Irf7, Mapk12, Ifih1, Tnf, Dhx58, Rnf125, Isg15	7
Apelin signaling pathway	Plin1, Sphk1, Mylk3, Serpine1, Gnb3, Spp1, Pik3r5, Gng11, Myl2, Rras, Gngt2	11
C-type lectin receptor signaling pathway	Nlrp3, Ptgs2, Mapk12, Bcl3, Tnf, Lsp1, Pycard, Cd209d, Rras, Clec4e	10
PI3K-Akt signaling pathway	Itgb4, Osmr, Col2a1, Col4a3, Fgf7, Epha2, Col4a4, Gh, Col6a1, Gnb3, Col9a3, Spp1, Pik3r5, Prl, Fgfr4, Eif4ebp1, Gng11, Itgb3, Gngt2, Il2rg, Pspn, Vwf	22
NOD-like receptor signaling pathway	Gsdmd, Irf7, Oas2, Nlrp3, Mapk12, Cyba, Tnf, Irgm2, Gbp2, Ccl12, Oas1a, Pycard, Naip5, Naip2, Gbp3, Gbp7, Irgm1	17
**Downregulated KEGG pathway**	**Downregulated genes**	
Calcium signaling pathway	Slc8a1, Atp2b3, Met, Nos2, Grin3a, Cacna1g, Ppp3r2, Drd5	8
Neuroactive ligand-receptor interaction	Htr1b, Nmbr, Chrna5, Htr1a, Mc5r, Grin3a, Npbwr1, Nts, Sstr4, Drd5	10
cAMP signaling pathway	Atp1a4, Atp2b3, Htr1b, Htr1a, Grin3a, Drd5	6
cGMP-PKG signaling pathway	Atp1a4, Slc8a1, Atp2b3, Irs1, Ppp3r2	5
Adrenergic signaling in cardiomyocytes	Atp1a4, Slc8a1, Atp2b3, Scn5a	4
