# Supplementary material for: Age- and ApoE Genotype-Dependent Transcriptomic Responses to O3 in the Hippocampus of Mice
Source: Int J Mol Sci. 2025 Mar 7;26(6):2407. doi: 10.3390/ijms26062407 (PMC11942628; doi:10.3390/ijms26062407)
Supplement: Supplementary file 1 [file ijms-26-02407-s001.zip › Supplementary Table S4c Ozone vs FA KEGG pathway.pdf]

**Supplementary Table S4c Ozone vs FA KEGG pathway**

| **Upregulated KEGG pathway** | **Upregulated genes** | |
|---|---|---|
| Neuroactive ligand-receptor interaction | Htr2c, Prlr, Glp1r, Glra2, Adra2a, Gabrq, Chrna3, Kng2, Adrb2, Lepr | 10 |
| Wnt signaling pathway | Fzd7, Tle4, Siah1b, Wnt8b, Sfrp5, Fzd4 | 6 |
| **Downregulated KEGG pathway** | **Downregulated genes** | |
| cAMP signaling pathway | Vav2, Creb3l3, Pomc, Lhcgr, Atp2a1, Oxt | 6 |
| Glucagon signaling pathway | Creb3l3, Pygm, Itpr1 | 3 |
| NF-kappa B signaling pathway | Tnfrsf11a, Ltb, Pias4 | 3 |
| Estrogen signaling pathway | Creb3l3, Itpr1, Pomc | 3 |
| Insulin signaling pathway | Ptpn1, Rhoq, Pygm | 3 |
| cGMP-PKG signaling pathway | Creb3l3, Itpr1, Atp2a1 | 3 |
| Prolactin signaling pathway | Tnfrsf11a, Lhcgr | 2 |
| Chemokine signaling pathway | Vav2, Ccl9, Ncf1 | 3 |
