# Supplementary material for: Age- and ApoE Genotype-Dependent Transcriptomic Responses to O3 in the Hippocampus of Mice
Source: Int J Mol Sci. 2025 Mar 7;26(6):2407. doi: 10.3390/ijms26062407 (PMC11942628; doi:10.3390/ijms26062407)
Supplement: Supplementary file 1 [file ijms-26-02407-s001.zip › Supplementary Table S5a 17M E4 vs E3 Ozone KEGG Pathway.pdf]

Supplementary Table S5a. E4_vs_E3_17M_Ozone_KEGG_pathway

TGF-beta signaling pathway	Thsd4, Fbn1, Nbl1, Tfrc, Bmp6, Hfe	6
Fatty acid elongation	Acaa2, Elovl7, Acot2	3
mTOR signaling pathway	Rnf152, Fzd7, Rraga, Prr5l, Wnt3, Wnt9a, Fzd6, Wnt8b, Cab39l	9
Adrenergic signaling in cardiomyocytes	Gnai1, Calml4, Ppp2r2a, Creb5, Plcb4, Cacnb2, Cacng8, Atp2b1, Adra1d	9
Oxytocin signaling pathway	Gnai1, Calml4, Nfatc2, Ptgs2, Cd38, Plcb4, Npr1, Cacnb2, Cacng8	9
Sphingolipid signaling pathway	Degs1, Gnai1, Abcc1, S1pr5, Sgms2, Ppp2r2a, Plcb4, Ctsd	8
Hippo signaling pathway	Fzd7, Lats2, Ywhaq, Ppp2r2a, Wnt3, Wnt9a, Bmp6, Fzd6, Ctnna1, Wnt8b	10
Wnt signaling pathway	Sfrp1, Fzd7, Nfatc2, Vangl1, Wnt3, Wnt9a, Fzd6, Plcb4, Wnt8b, Prickle3, Sfrp5	11
Calcium signaling pathway	Fgf10, Htr2c, Calml4, Nfatc2, Met, Stim2, Itpkb, Cd38, Fgfr2, Sln, Plcb4, Adora2a, Atp2b1, Adra1d	14
**Downregulated KEGG pathway**	**Downregulated genes**	
ErbB signaling pathway	Shc3, Pak6, Plcg2, Mapk3, Jun, Cdkn1a Elk1, Camk2d	8
Oxytocin signaling pathway	Ryr1, Kcnj5, Kcnj14, Mapk3, Jun, Cdkn1a, Adcy1, Elk1, Camk2d Oxt	10
GnRH signaling pathway	Mmp14, Map2k6, Mapk3, Jun, Adcy1, Elk1, Camk2d	7
Calcium signaling pathway	Tacr3, Ryr1, Trhr, Cacna1h, Trdn, Plcg2, Hrh1, Mst1, Atp2b4, Adcy1, Nos1, Camk2d	12
cAMP signaling pathway	Ppara, Adcyap1r1, Mapk3, Jun, Sstr1, Ghrl, Tiam1, Atp2b4, Adcy1, Camk2d, Oxt	11
Ras signaling pathway	Pla2g3, Kitl, Shc3, Pak6, Rasa3, Plcg2, Mapk3, Tiam1, Elk1, Mras, Gngt2	11
Neuroactive ligand-receptor interaction	Pth2r, Drd4, Tacr3, Trhr, Grm2, Adcyap1r1, Hrh1, Calca, Sstr1, Ghrl, Nts, Kiss1r, Pth1r, Adra2c, Oxt	15
Apelin signaling pathway	Ryr1, Myl3, Smad3, Mapk3, Adcy1, Mras, Gngt2, Nos1	8
mTOR signaling pathway	Wnt7a, Wnt7b, Wnt4, Mapk3, Wnt5a, Ulk1, Ulk2, Deptor	8
Relaxin signaling pathway	Shc3, Col4a2, Mapk3, Jun, Adcy1, Gngt2, Nos1	7
Wnt signaling pathway	Wnt7a, Wnt7b, Smad3, Wnt4, Jun, Wnt5a, Rspo2, Camk2d	8
C-type lectin receptor signaling pathway	Plcg2, Mapk3, Jun, Il17d, Mras, Casp1	6
Phospholipase D signaling pathway	Kitl, Shc3, Grm2, Plcg2, Mapk3, Adcy1, Mras	7
Thyroid hormone signaling pathway	Dio3, Wnt4, Plcg2, Mapk3, Med27, Dio2	6
Adrenergic signaling in cardiomyocytes	Tpm2, Myl3, Mapk3, Atp2b4, Adcy1, Tnnt2, Camk2d	7
Estrogen signaling pathway	Shc3, Kcnj5, Mapk3, Jun, Adcy1, Esr2	6
Apelin signaling pathway	Ryr1, Myl3, Smad3, Mapk3, Adcy1, Mras, Gngt2, Nos1	8
AGE-RAGE signaling	Smad3, Col4a2, Plcg2, Mapk3, Jun	5

pathway in diabetic complications		
p53 signaling pathway	Sesn3, Apaf1, Cdkn1a, Sesn1	4
HIF-1 signaling pathway	Plcg2, Mapk3, Cdkn1a, Hk2, Camk2d	5
TNF signaling pathway	Mmp14, Map2k6, Lif, Mapk3, Jun	5
Hippo signaling pathway	Wnt7a, Wnt7b, Smad3, Wnt4, Wnt5a, Dlg5	6
Neurotrophin signaling pathway	Shc3, Plcg2, Mapk3, Jun, Camk2d	5
