# Supplementary material for: Age- and ApoE Genotype-Dependent Transcriptomic Responses to O3 in the Hippocampus of Mice
Source: Int J Mol Sci. 2025 Mar 7;26(6):2407. doi: 10.3390/ijms26062407 (PMC11942628; doi:10.3390/ijms26062407)
Supplement: Supplementary file 1 [file ijms-26-02407-s001.zip › Supplementary Table S5b 17M E4 vs E3 Ozone Hallmark Pathway.pdf]

Supplementary Table S5b. 17M E4 vs E3 Ozone Hallmark Pathway

**Upregulated Hallmark pathway**	**Genes**	**Count**
Oligodendrocyte_markers	Actc1, Serpinb1c, Tmem88b, Mag, Gpr37, Mog, Cnp, S1pr5, Ermn, Fa2h, Tmem63a, Mal, Tspan2, Mobp, Anln, Sgk1, Tppp3, Myrf, Sox10, Serpinb1a, Opalin, Mcam, Gjb1, Ttyh2, Plekhg3, Gatm, Clic4, Mbp, Ugt8a, Gsn, Serinc5, Cers2, Plekhb1, Rnf13, Car2, Slc44a1, Qdpr, Arhgef10, Olig1, Olig2, Omg, Arrdc3, Gltp, Apod, Wscd1, Taldo1, Ndrg1	47
Eicosanoid metabolism via Lipoxygenases lox	Dpep1, Dpep2, Alox15, Acox2, Ltc4s, Ehhadh, Alox5, Ggt5, Mgst2, Ptgr1, Cyp4a12b, Alox12	12
Hevner telencephalon choroid plexus epithelial cells	Ttr, Cldn2, Sostdc1, Aqp1, Krt18, Calml4, Krt8, Msx1	8
**Downregulated** Hallmark **pathway**	**Genes**	
Reactome role of lat2 ntal lab on calcium mobilization	Igkv2-137, Ighv8-12, Igkv1-99, Igkv1-117, Ighv5-2, Ms4a2, Igkv1-122, Igkv17-121, Fcer1a, Iglc2, Igkv1-110, I gll1	12
Reactome fceri mediated NF KB activation	Igkv2-137, Ighv8-12, Igkv1-99, Igkv1-117, Uba52, Ighv5-2, Ms4a2, Igkv1-122, Igkv17-121, Fcer1a, Iglc2, Igkv1-110, Igll1, Psmb11, Ube2v1, Psma8	16
Reactome cell surface interactions at the vascular wall	Sell, Sirpb1c, Igkv2-137, Trem1, Angpt4, Igha, Ighv8-12, Igkv1-99, Igkv1-117, Cd44, Itgal, Dok2, Gm5150, Ighv5-2, Mmp1a, Igkv1-122, Igkv17-121, Sirpb1b, Iglc2, Igkv1-110, Grb7, Igll1, Slc16a3, Lck, Procr, Slc7a11, Fn1, Slc7a9, Angpt1, Itga4, Mertk, Itga3, Angpt2	33
Reactome gap junction trafficking and regulation	Tubb1, Gja8, Gjb5, Gjb3, Gjd4, Gja10, Gjb4, Tuba3a, Gja5	9
Hevner subventricular zone and up neuron fate committed cells	Nnat, Sox11, Gpc2, Tubb3, D430019H16Rik, Elavl4, Ulk1, Palm, Dpysl5, Dtx1, Elavl3, Dpf1, Mmp24, Apc2, Pik3r3, Phf21b, Syt7, Dcc	18
Reactome_antigen_activates_b_cell_receptor_bcr_leading_to_generation_of_second_messengers	Igkv2-137, Ighv8-12, Igkv1-99, Igkv1-117, Ighv5-2, Igkv1-122, Igkv17-121, Iglc2, Igkv1-110, Cd79a, Igll1, Plcg2, Syk, Itpr3, Btk	15
