# Supplementary material for: Age- and ApoE Genotype-Dependent Transcriptomic Responses to O3 in the Hippocampus of Mice
Source: Int J Mol Sci. 2025 Mar 7;26(6):2407. doi: 10.3390/ijms26062407 (PMC11942628; doi:10.3390/ijms26062407)
Supplement: Supplementary file 1 [file ijms-26-02407-s001.zip › Supplementary Table S6 List of qRT-PCR primers.pdf]

Supplementary Table S6. List of qRT-PCR primers

Gene	Sequence
Barhl1	For: CAAAGTGAAGGAGGAGGGCG
Barhl1	Rev: GTGTCGGTGAGGTTGAGCGA
Twist1	For: GCT TGA GGG TCT GAA TCT TGC T
Twist1	Rev: GTC CGC AGT CTT ACG AGG AG
Bok	For: 5'-CAAGGTGGTGTCCCTGTATG-3'
Bok	Rev: 5'-GAATAAACCCTGGAAGTGGTG-3'
Sfrp5	For: 5'-ATGCTGCACTGCCACAAGTTC 3'
Sfrp5	Rev: 5'-TGCGCATCTTGACCACAAAG 3'
Klotho	For: 5′-AGACCTCCCGATGTATGTGAC-3′
Klotho	Rev: 5′-CGAGATGAAGACCAGCAAAG-3′
Pon3	For: 5′-GATCTGAATGAGCAAAACCCAGAGGC-3′
Pon3	Rev: 5′-GAGTCCATGTTGGGGTGATTCACGAC-3′
Folr1	For: 5′-ATTCCTTGGTGCCACTGACC-3′
Folr1	Rev: 5′-ATAGAACCTCGCCACCTCCT-3′
Casp1	For: 5'-CAGACAAGGGTGCTG AACAA-3'
Casp1	Rev: 5'-TCGGAATAACGGAGTCAATCA-3'
DC	For: 5'-GAGTGGGGCTTTCGAGTGAT-3'
DC	Rev: 5'-GGTGGAACCACAGCAACTTTT-3'
NeuN	For: 5'-GGCAATGGTGGGACTCAAAA-3'
NeuN	Rev: 5'-GGGACCCGCTCCTTCAAC-3'
